# A novel statistical methodology for quantifying the spatial arrangements of axons in peripheral nerves

**DOI:** 10.3389/fnins.2023.1072779

**Published:** 2023-03-09

**Authors:** Abida Sanjana Shemonti, Emanuele Plebani, Natalia P. Biscola, Deborah M. Jaffey, Leif A. Havton, Janet R. Keast, Alex Pothen, M. Murat Dundar, Terry L. Powley, Bartek Rajwa

**Affiliations:** ^1^Department of Computer Science, Purdue University, West Lafayette, IN, United States; ^2^Department of Computer & Information Sciences, Indiana University - Purdue University Indianapolis, Indianapolis, IN, United States; ^3^Department of Neurology, Icahn School of Medicine at Mount Sinai, New York, NY, United States; ^4^Department of Psychological Sciences, Purdue University, West Lafayette, IN, United States; ^5^Department of Neuroscience, Icahn School of Medicine at Mount Sinai, New York, NY, United States; ^6^James J. Peters Department of Veterans Affairs Medical Center, Bronx, NY, United States; ^7^Department of Anatomy and Physiology, University of Melbourne, Melbourne, VIC, Australia; ^8^Bindley Bioscience Center, Purdue University, West Lafayette, IN, United States

**Keywords:** peripheral nervous system, neuroanatomy, neuromodulation, spatial point process, optimal transport problem, Sinkhorn distance

## Abstract

A thorough understanding of the neuroanatomy of peripheral nerves is required for a better insight into their function and the development of neuromodulation tools and strategies. In biophysical modeling, it is commonly assumed that the complex spatial arrangement of myelinated and unmyelinated axons in peripheral nerves is random, however, in reality the axonal organization is inhomogeneous and anisotropic. Present quantitative neuroanatomy methods analyze peripheral nerves in terms of the number of axons and the morphometric characteristics of the axons, such as area and diameter. In this study, we employed spatial statistics and point process models to describe the spatial arrangement of axons and Sinkhorn distances to compute the similarities between these arrangements (in terms of first- and second-order statistics) in various vagus and pelvic nerve cross-sections. We utilized high-resolution transmission electron microscopy (TEM) images that have been segmented using a custom-built high-throughput deep learning system based on a highly modified U-Net architecture. Our findings show a novel and innovative approach to quantifying similarities between spatial point patterns using metrics derived from the solution to the optimal transport problem. We also present a generalizable pipeline for quantitative analysis of peripheral nerve architecture. Our data demonstrate differences between male- and female-originating samples and similarities between the pelvic and abdominal vagus nerves.

## 1. Introduction

Understanding the functionalities of the peripheral nerves and developing neuromodulation tools require an in-depth quantitative characterization of the anatomy of the nerves. A large portion of the quantitative neuroanatomical studies focus on counting and comparing the number of myelinated and unmyelinated axons in the peripheral nerves in different animals (Hoffman and Schnitzlein, 1961; Krous et al., 1985; Asala and Bower, 1986; Prechtl and Powley, 1990; Pereyra et al., 1992; Soltanpour and Santer, 1996; Safi et al., 2016). There are studies on analyzing the changes in the number of myelinated and unmyelinated axons as the function of animals' age (Krous et al., 1985; Pereyra et al., 1992; Soltanpour and Santer, 1996). The morphometric characteristics of the axons, such as area of axon cross-section, diameter, myelin thickness, are also well-developed and helpful for estimating electrode distances for neuromodulation purposes (Asala and Bower, 1986; Prechtl and Powley, 1990; Walter and Tsiberidou, 2019; Pelot et al., 2020; Havton et al., 2021; Settell et al., 2021).

The vagus is a complex, multi-functional peripheral nerve of the autonomic nervous system, containing both sensory and motor axons that regulate a wide variety of functions (Câmara and Griessenauer, 2015; Breit et al., 2018). These include regulation of the heart, respiratory tract, and many areas of the gastrointestinal system, influencing motility, secretions, and communication with the immune system. This breadth of activity and its bidirectional connectivity with the central nervous system have led to the vagus becoming a promising target for bioelectric medicine, through development of specific protocols for vagal nerve stimulation (VNS) (Bonaz et al., 2017a,b; Horn et al., 2019).

In addition to providing an alternative therapeutic approach for drug-resistant clinical conditions within organs, the vagal afferent connections to the brain provide opportunities for novel therapies directed to various psychiatric disorders. To improve the efficacy and specificity of VNS for each type of clinical condition, a greater understanding of the intra-vagal neural elements relating to each organ system is required (Howland, 2014; Thompson et al., 2019, 2023), as demonstrated by a recent study showing that fascicle-selective stimulation can reduce off-target effects of VNS (Thompson et al., 2023). This includes understanding the spatial organization of different functional classes of axons within and between fascicles. This spatial organization has not been investigated in depth within the visceral nervous system.

We have begun to address this knowledge gap using an extensive dataset of transmission electron microscopy (TEM) images derived from multiple cross-sections of the rat vagus. We have included in our study additional TEM images from the rat pelvic nerve, another multi-functional major nerve of the autonomic nervous system that supplies sensory and motor axons to the urogenital organs and lower bowel. Both TEM data sets have been published through the SPARC Portal RRID:SCR_017041 under a CC-BY 4.0 license (Plebani et al., 2022).

Cross-sections of large peripheral nerves reveal a variety of components (myelinated and unmyelinated axons, Schwann cells), high-order structures (fascicles and Remak bundles), and raise questions regarding the spatial arrangement of these components, similarities between multiple arrangements, and their relationship to various biological factors including age, sex, and diseases. This study focuses on the unmyelinated axons segmented utilizing our high-throughput deep learning model (Plebani et al., 2022). We aim to define a notion of similarity (or dissimilarity) between the spatial arrangements of the unmyelinated axons and quantify the distances between them. We resort to spatial point patterns to represent the image data conveniently for analyzing the axons' spatial organization. We use the centroids of the segmented axons to construct spatial point patterns. We consider spatial inhomogeneity and anisotropy to be the spatial features to represent the spatial arrangement of the axons, and be used for quantification. A known way to get an intuitive sense of spatial inhomogeneity (inhibition and/or attraction between points) and anisotropy of a point pattern is to investigate its second-order statistics (Ripley, 1976, 1977; Sengupta et al., 2013; Dixon, 2014). Since Ripley's summary of spatial statistical methods in 1977 the techniques for spatial pattern analysis have been occasionally employed in neuroscience, often by statisticians who saw the extraordinary complexity of neuroanatomical patterns to be a perfect demonstration of the spatial statistics inference ability (Bjaalie et al., 1991; Diggle et al., 1991; Prodanov et al., 2007; Jafari-Mamaghani et al., 2010; Waller et al., 2011).

Although the standard spatial statistical measures can quantify overall global differences between point interactions, they are not well suited for calculating distances between complex non-random patterns with multiple distinct local interactions. Therefore, we propose a method that involves computing the local second-order spatial statistics for the nerve fascicles to capture their spatial arrangement and utilizing a revised optimal transport distance (Sinkhorn distance) to measure similarities between the second-order spatial statistics of every pair of nerve cross-sections. We visualize the resulting Sinkhorn distance matrix in a new metric space using multi-dimensional scaling that helps interpret the similarity (dissimilarity) of the spatial features in the nerve cross-sections. Our concept of Sinkorn distance embedding was influenced by related work on optimal transport-based morphometry applications in cell biology (Wang et al., 2013; Basu et al., 2014).

In addition to addressing a neuroscientific problem of quantifying the vagus nerve anatomy with computer science tools, we intend to bring together a variety of approaches from various computer science domains to extend the toolkit for point-pattern comparisons in biology. Utilizing the optimal transport framework to establish a quantitative measure for spatial point patterns and advance the workflow of quantitative analysis of peripheral nerve architecture, our method could be used beyond neuroscience. We believe that our findings contribute to the establishment of spatially selective stimulation of nerve axons to improve the efficacy of VNS.

**Figure 1:**
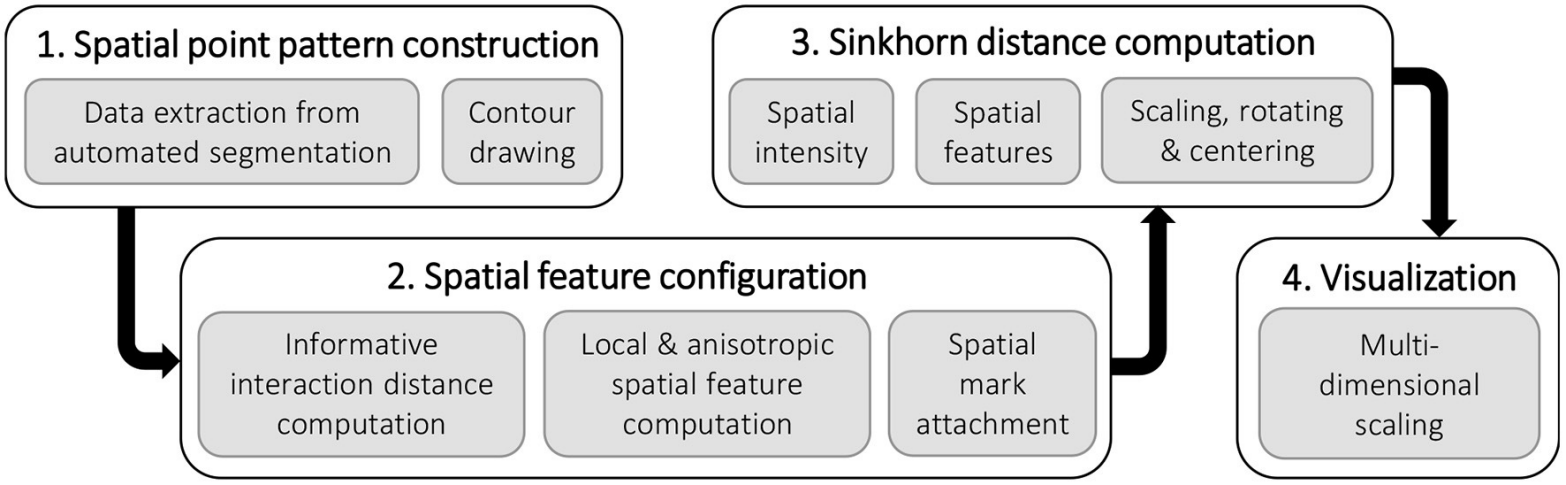
The pipeline for the quantitative analysis of the spatial arrangement of axons in the peripheral nerve cross-sections.

Figure 1 depicts a high-level overview of the steps in the quantitative analysis of the spatial arrangement of axons in the peripheral nerve architecture presented in this paper. We explain each component of the pipeline in the following sections. The biological data and the data preprocessing steps are described in Section 2.1. The basics of spatial point pattern, spatial statistics, and optimal transport framework are introduced in Section 2.2 and Section 2.3, respectively. We provide details on the experimental setup and results in Section 3, which cover steps 2, 3, and 4 of the computational pipeline. We discuss the results of the empirical study in Section 4 before concluding.

## 2. Materials and methods

### 2.1. Biological data and automated segmentation

**Figure 2:**
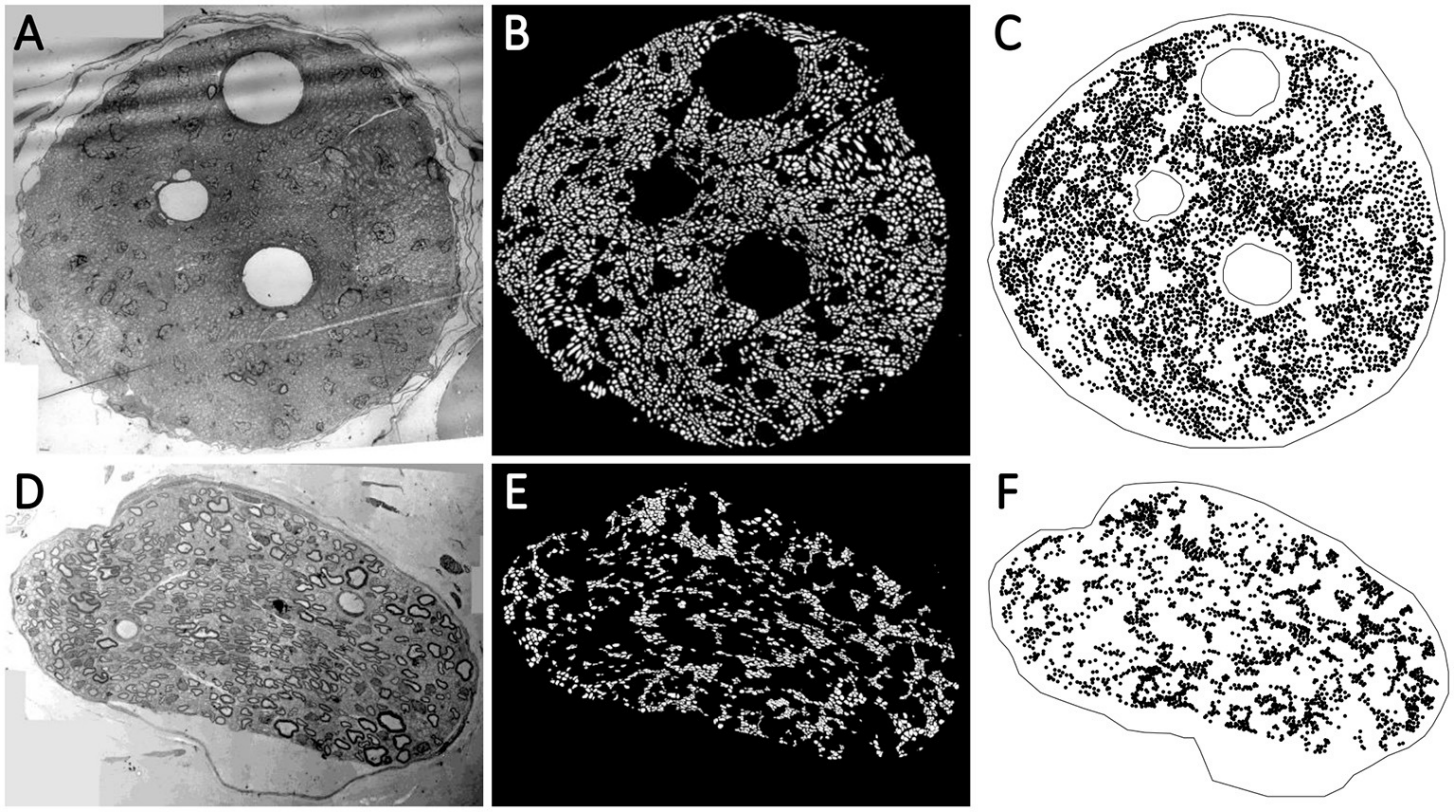
**(A, D)** The transmission electron microscopy (TEM) images of the nerve cross-section of Image 15 (vagus) and Image 29 (pelvic) listed in Table 1, respectively. The visible void spaces in the nerve cross-sections are blood vessels. The tiny light gray regions without any border are the unmyelinated axons. The myelinated axons have slightly darker gray borders. **(B, E)** The automated segmentation of the unmyelinated axons (the white regions) in the nerve cross-sections. **(C, F)** The spatial point patterns constructed with the centroid locations (the black circles) of the segmented unmyelinated axons.

Although the vagus nerve anatomy was the primary motivation behind this study, we use the TEM images of the vagus and pelvic nerve cross-sections in rats for comparisons. A list of the TEM images used in this study is shown in Table 1. The protocols and techniques followed for nerve sample collection, processing, and imaging are documented in our previous work (Plebani et al., 2022). The data is publicly available *via* NIH-supported SPARC Pennsieve database (Havton et al., 2022). Briefly, the unmyelinated axons in some of these TEM images were manually annotated and used as labeled data to train, validate, test, and evaluate an automated segmentation model based on the U-Net architecture (Ronneberger et al., 2015; Plebani et al., 2022). The segmentation model is a U-Net with four stages: the convolutional layers have a batch normalization layer followed by a ReLU activation layer, and the bottleneck stage has extra dropout layers between convolutions (Plebani et al., 2022). The model classifies the TEM image pixels as one of the three following classes: (a) *fiber* if it is inside an unmyelinated axon, (b) *border* if it is in a boundary region between an axon and the rest of the image defined by the outer edge of each axon, and (c) *background*. An updated version of the model^1^ was used here to segment the unmyelinated axons. The resulting axon counts are listed in Table 1. We used the open-source image processing package Fiji (Schindelin et al., 2012) to extract the centroid coordinates of the segmented unmyelinated axons and the functions in R packages to establish the outer boundaries and inner void spaces of the nerve cross-sections, to construct the spatial point patterns. Images 15 (vagus) and 29 (pelvic) listed in Table 1 are shown in Figures 2A, D, respectively, along with their corresponding automated segmentations and spatial point patterns.

**Table 1:** The list of the TEM images of vagus and pelvic nerve cross-sections in rats used in this study.

**Image ID**	**Image size** **(pixel × pixel)**	**Resolution** **(nm/pixel)**	**Nerve**	**Location**	**Sex**	**No. of segmented** **axons**
1	2,994 × 2,497	11.9	Vagus	Right CT	F	183
2	10,624 × 6,686	11.9	Vagus	Right CT	F	5,020
3	21,005 × 22,847	11.9	Vagus	Right CT	F	13,375
4	7,707 × 7,978	11.9	Vagus	AVAT	F	4,538
5	9,633 × 15,046	11.9	Vagus	AVPT	F	10,328
6	13,120 × 14,400	11.9	Vagus	AVAG	F	6,566
7	19,921 × 9,680	8.7	Vagus	AVPT	F	8,980
8	5,175 × 3,784	11.9	Vagus	AVAG	F	407
9	12,328 × 9,692	13.7	Vagus	Right CT	M	7,647
10	6,794 × 5,472	13.7	Vagus	Right CT	M	871
11	5,262 × 7,111	13.7	Vagus	AVPT	M	9,109
12	24,746 × 20,682	11.9	Vagus	Right CT	F	12,992
13	20,372 × 27,269	11.9	Vagus	Left CT	F	14,155
14	7,953 × 5,781	11.9	Vagus	AVAG	F	1,698
15	8,446 × 7,258	13.7	Vagus	AVAG	M	4,938
16	4,128 × 4,068	13.7	Vagus	AVAG	M	1,061
17	9,935 × 8,870	13.7	Vagus	AVAG	M	4,654
18	5,521 × 4,971	13.7	Vagus	AVAG	M	1,409
19	8,633 × 8,866	11.9	Pelvic	≤ 2 mm from PG	M	1,663
20	3,891 × 3,334	11.9	Pelvic	≤ 2 mm from PG	M	297
21	2,754 × 2,958	11.9	Pelvic	≤ 2 mm from PG	M	209
22	3,357 × 3,823	11.9	Pelvic	≤ 2 mm from PG	M	303
23	4,419 × 5,701	11.9	Pelvic	≤ 2 mm from PG	M	608
24	2,804 × 4,221	11.9	Pelvic	≤ 2 mm from PG	M	350
25	5,064 × 7,207	11.9	Pelvic	≤ 2 mm from PG	M	652
26	5,869 × 6,268	11.9	Pelvic	≤ 2 mm from PG	M	990
27	7,941 × 6,372	11.9	Pelvic	≤ 2 mm from PG	M	1,372
28	4,028 × 3,513	11.9	Pelvic	≤ 2 mm from PG	M	460
29	11,129 × 7,962	11.9	Pelvic	≤ 2 mm from PG	M	2,363

The vagus and the pelvic nerve cross-sections are collected from the following nerve locations. CT, cervical trunk; AVAT, abdominal vagus anterior trunk; AVPT, abdominal vagus posterior trunk; AVAG, abdominal vagus anterior gastric (ventral gastric branch); PG, pelvic ganglion. The information regarding the Images 12–29 were published in Plebani et al. (2022).

Before reporting the experimental details, we provide a brief overview of spatial point patterns, spatial statistics concepts, and optimal transport framework in the following two subsections.

### 2.2. Spatial point patterns and spatial statistics

**Figure 3:**
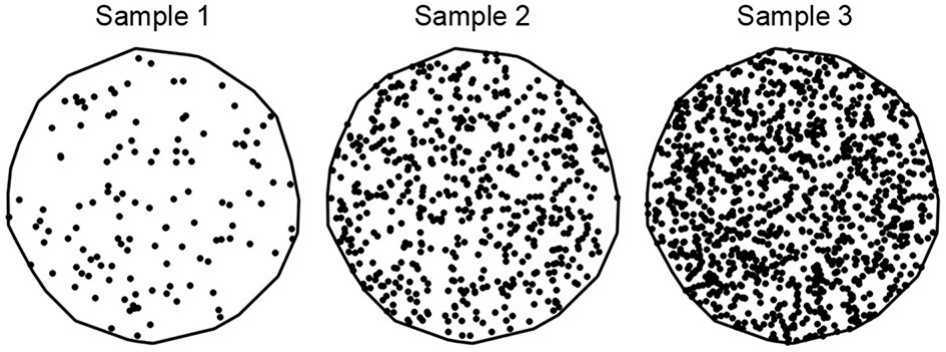
An illustration of spatial point patterns with different spatial intensities. Samples 1, 2, and 3 have 20, 100, and 200 points per unit area, respectively.

A *spatial point pattern* (SPP) is a set of spatial locations associated with entities of interest in 2-D or 3-D space, encompassed by an observation window (Møller and Waagepetersen, 2003; Stoyan, 2006; Jafari-Mamaghani et al., 2010; Baddeley et al., 2015). The objective of SPP analysis is to examine the spatial arrangement of the points in an SPP and recognize trends that define the point pattern. Two fundamental descriptive characteristics of an SPP are *intensity* and *interaction*. The intensity ρ or ρ(*u*) of a point pattern, a first-order statistics, is the average number of points per unit area, and it can be uniform across the observation window (homogeneous), or it can vary according to an intensity function (inhomogeneous). A point pattern's intensity is usually denoted by λ in literature, but we use ρ to avoid confusion with another notation related to the optimal transport problem. Three SPPs of different average intensity, with 20 (sample 1), 100 (sample 2), and 200 (sample 3) points per unit area are illustrated in Figure 3.

The interaction is associated with a distance *r* and describes the influence the points have on their neighbors within *r* radius. The interaction is termed complete spatial randomness (CSR) if the points are independent. The points can exhibit positive interaction (spatial attraction), negative interaction (spatial inhibition), or a combination of both.

It is common practice to use second-order statistics such as Besag's centered *L*-function, which is a transformation of Ripley's *K*-function (Ripley, 1976, 1977; Besag, 1977), to investigate the interaction in point patterns. Let **X** be a point pattern and *t*(*u, r*, **X**) be the number of points in **X** which lie within distance *r* of the location *u*. Assuming **X** is a homogeneous point pattern with intensity ρ, the number of points within distance *r* of a specific point is represented by ρ*K*(*r*) (Ripley, 1976).


(1) $$\begin{array}{l} \text{Ripley's }K\text{-function}:K\left(r\right)\text{=}\frac{𝔼\left[t\left(u,r,X\right)\text{|}u\in X\right]}{\rho },\\ \text{Besag's centered }L\text{-function}:L(r)\text{=}\sqrt{\frac{K\left(r\right)}{\pi }}-r \end{array}$$

An estimator for the empirical *K*-function $\hat{K}\left(r\right)$ is formulated in Equation (2), and represents the cumulative average number of neighbors within *r* radius of a typical point, standardized by the intensity and corrected for edge effects.


(2) $$\begin{array}{l} \hat{K}\left(r\right)=\frac{W}{n\left(n-1\right)}\overset{n}{\underset{i=1}{\sum }}\overset{n}{\underset{j=1,j\neq i}{\sum }}\nu \left(d_{ij}⩽r\right)e_{ij}\left(r\right) \end{array}$$

where ν(.) is an indicator function that equals 1 if the argument is true and otherwise is 0. Here *n* is the number of points; *W* is the area of the observation window; *r* is the interaction distance; *d*_*ij*_ is the Euclidean distance between *x*_*i*_ and *x*_*j*_; and *e*_*ij*_ denotes weights for edge correction (Baddeley et al., 2015). The *K*- and *L*-functions can illustrate the non-random spatial arrangement of the points if compared with CSR. They are invariant to the intensity of a point pattern and to missing random points (Ripley, 1976; Baddeley et al., 2000), which allows these second-order statistics to be compared when the number of points and observation window vary in the point patterns under consideration. Positive values of the centered *L*-function depict spatial attraction, and negative values describe spatial inhibition in a point pattern.

**Figure 4:**
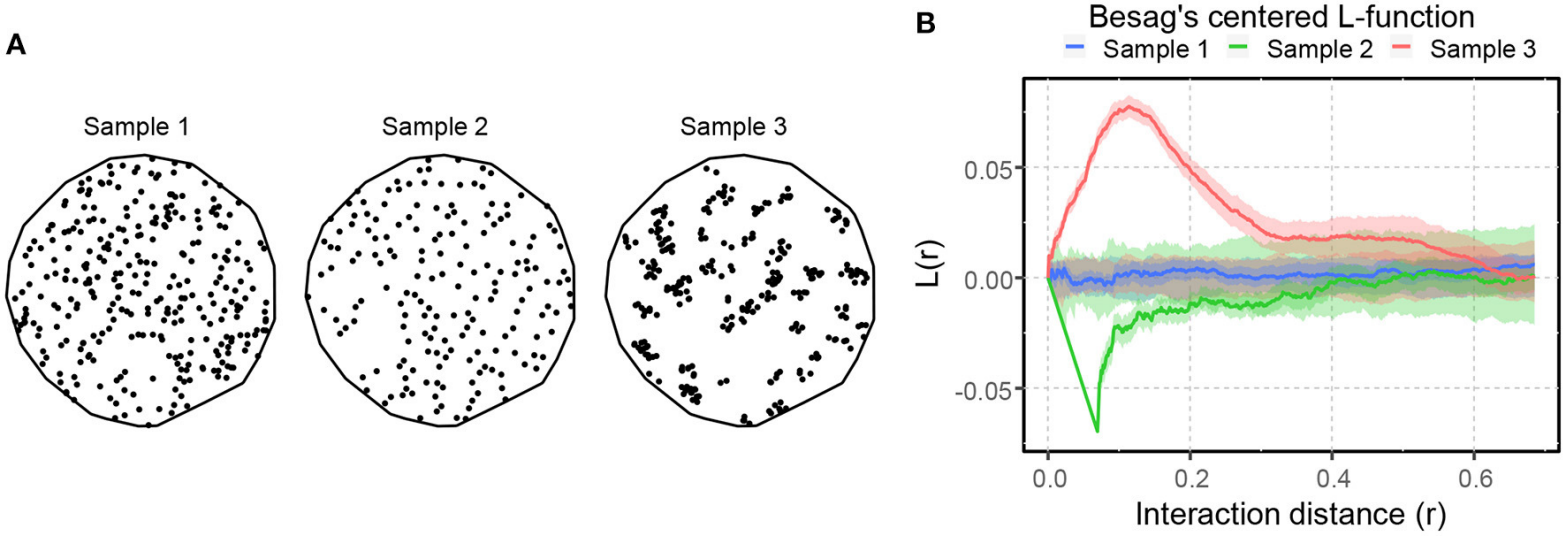
An illustration of spatial point patterns with different spatial interactions. **(A)** SPPs portraying complete spatial randomness (sample 1), spatial inhibition (sample 2), and spatial attraction (sample 3). **(B)** Besag's centered *L*-function computed for the patterns shown in **(A)**. The solid lines illustrate the spatial interaction of the patterns compared to CSR. The shaded area around the solid lines shows the boundaries of 95-percentile confidence interval.

When dealing with inhomogeneous point patterns, an inhomogeneous *L*-function, based on the inhomogeneous *K*-function *K*_inhom_(*r*) can be evaluated. The estimator for the inhomogeneous *K*-function ${\hat{K}}_{\text{inhom}}\left(r\right)$ is formulated in Equation (3) below.


(3) $$\begin{array}{l} {\hat{K}}_{\text{inhom}}\left(r\right)=\frac{1}{D^{p}W}\sum_{i=1}^{n}\sum_{j=1,j\neq i}^{n}\frac{\nu (d_{ij}⩽r)}{\hat{\rho }\left(x_{i}\right)\hat{\rho }\left(x_{j}\right)}e_{ij}(r),\\ D^{p}={\left(\frac{1}{W}\sum_{i=1}^{n}\frac{1}{\hat{\rho }\left(x_{i}\right)}\right)}^{p},\;p\in \left\{1,2\right\} \end{array}$$

where $\hat{\rho }\left(u\right)$ is an estimator of the intensity function ρ(*u*), obtained using a kernel-smoothed (described later in the paper) intensity estimator (Baddeley et al., 2015). Figure 4A shows three SPPs portraying complete spatial randomness (Sample 1), spatial inhibition (Sample 2), and spatial attraction (Sample 3). These point patterns were formed by the Poisson process, the hardcore process, and the Matern cluster process (Baddeley et al., 2015). The *L*-function illustrates the differences in spatial interaction of these SPPs, as shown in Figure 4B. The blue curve's fluctuation about zero within its significance band indicates that the point pattern in Sample 1 is most likely random, but the green and orange curves' prominent negative and positive peaks imply spatial inhibition and attraction in Samples 2 and 3, respectively.

**Figure 5:**
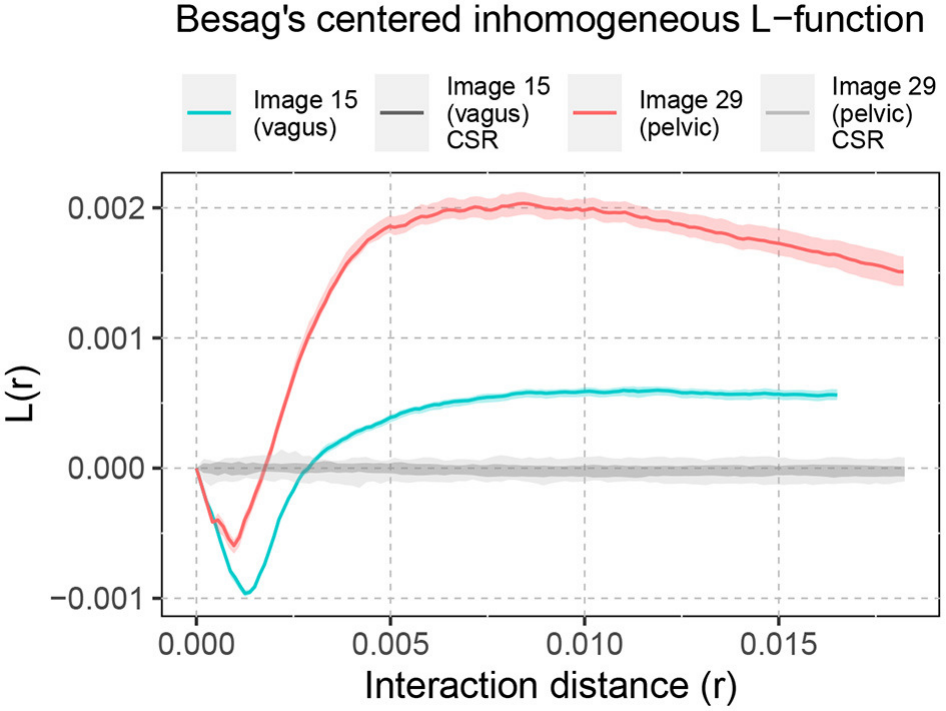
Besag's centered inhomogeneous *L*-function computed for Images 15 and 29. The shaded area around *L*(*r*) = 0 shows the significance bands of complete spatial randomness (CSR). The solid lines illustrate the non-random spatial arrangement of the point patterns compared to CSR. The shaded area around the solid lines shows the boundaries of 95-percentile confidence interval.

Similar analysis of spatial interaction can be done for the biological point patterns as well. Figure 5 shows the Besag's centered inhomogeneous *L*-function computed for Image 15 (vagus) and Image 29 (pelvic), listed in Table 1 and displayed in Figure 2. For both samples, this demonstrates spatial inhibition over a narrow interaction distance range, followed by spatial attraction. The clustering tendency (spatial attraction) is more pronounced in the pelvic sample.

**Figure 6:**
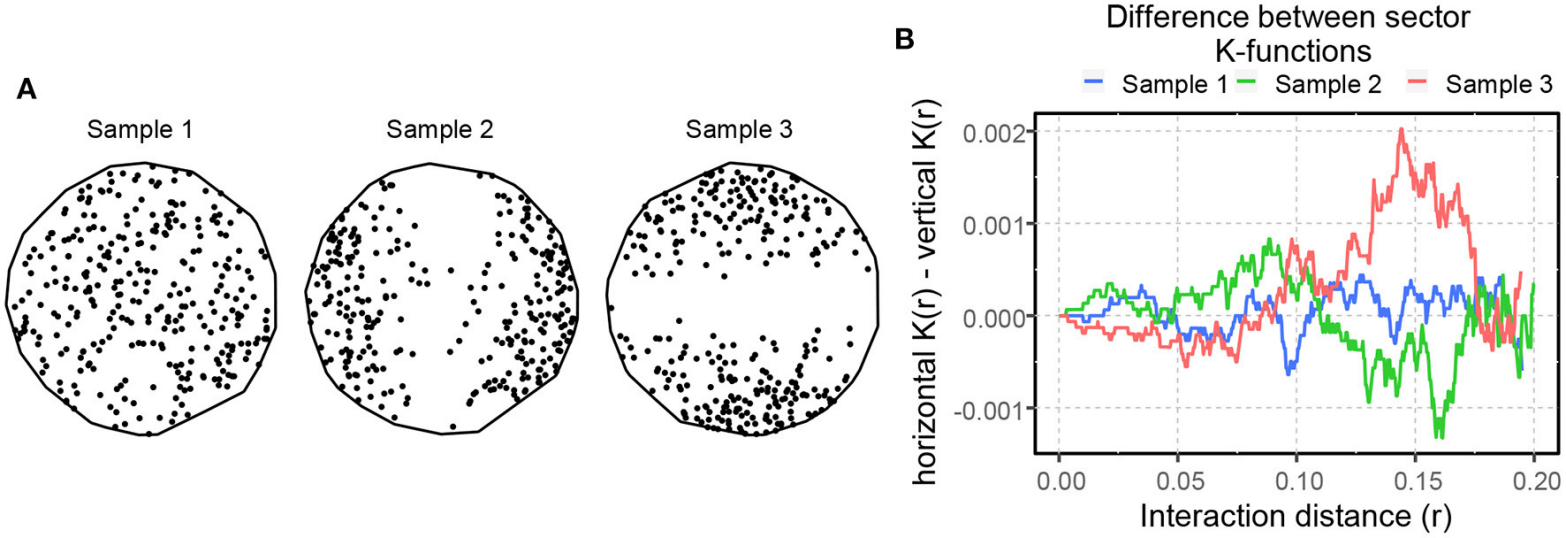
**(A)** Inhomogeneous random spatial point patterns with no directional preference (Sample 1), horizontal (Sample 2), and vertical (Sample 3) directional preferences. **(B)** Differences computed between the horizontal and vertical *K*-functions for the samples in **(A)**.

An SPP is *anisotropic* if any of its statistical characteristics change when the point pattern is rotated about any axis in 2-D or 3-D space. The *K*- and *L*-functions can be modified in various ways to estimate anisotropy (Ohser and Stoyan, 1981; Chiu et al., 2013). Computing the cumulative distribution of the neighbors within a section of the disc of *r* radius between two directional preferences θ_1_ and θ_2_, instead of the entire disc of *r* radius, gives the sector *K*- and *L*-functions (Baddeley et al., 2015). Figure 6A depicts three random point patterns with no preferential direction (Sample 1) as well as horizontal (Sample 2) and vertical anisotropies (Sample 3). We compute sector *K*-functions for sectors forming a 15° segment around the horizontal (0°) and vertical (90°) axes, and if these two functions are not approximately equal, we conclude that the point patterns are anisotropic (Baddeley et al., 2015). Figure 6B shows the differences between the horizontal and vertical *K*-functions for the three aforementioned point patterns. We observe that the difference between the sector *K*-functions fluctuates around zero for Sample 1, indicating the absence of anisotropy, holds positive values for Sample 2 and negative values for Sample 3, indicating horizontal and vertical preferences, respectively, up to a certain interaction distance.

When an SPP exhibits different interactions in different places, it is beneficial to look into the spatial statistics locally by decomposing them into contributions from individual points (Baddeley et al., 2015). If the *K*-function estimator $\hat{K}\left(r\right)$ shown in Equation (2) is decomposed, the contributions from individual points are referred to as local *K*-functions and can be formulated as follows:


(4) $$\begin{array}{l} \hat{K}\left(r,x_{i}\right)=\frac{W}{n-1}\overset{n}{\underset{j=1,j\neq i}{\sum }}\nu \left(d_{ij}⩽r\right)e_{ij}\left(r\right),\text{ for }i=1,\ldots ,n \end{array}$$

The estimator $\hat{K}\left(r\right)$ is simply the average of all the $\hat{K}\left(r,x_{i}\right)$s for *i* = 1, …, *n*. The centered local *L*-functions are formulated as $\hat{L}\left(r,x_{i}\right)={\left(\hat{K}\left(r,x_{i}\right)/\pi \right)}^{1/2}-r$. This notion of decomposition is applicable for the inhomogeneous and anisotropic *K*- and *L*-functions as well.

The points in an SPP may be of different types (multitype point pattern). The additional information attached to each point in point patterns is called a mark and can hold categorical or continuous-valued, physical or statistical characteristics. The points can carry additional attributes (forming marked point patterns) or be linked to the space of interest (covariates). It is often helpful to apply spatial smoothing to the marks of a point pattern for visualization and various post-processing purposes. The result of the kernel-smoothing (usually with Gaussian kernel) at a location *u* is a spatially weighted average of the marks attached to the points in the neighborhood of *u* (Baddeley et al., 2015). This is also known as the Nadaraya-Watson smoother (Nadaraya, 1964, 1989; Watson, 1964).

There are several partially similar, but differently interpreted spatial statistical functions employed to describe the dependence (*K*-function, *L*-function, pair correlation function) and the spacing (nearest-neighbor function *G*, empty-space function *F*, and their combination called the *J*-function) between points in an SPP (Baddeley et al., 2015). Although we used local inhomogeneous and anisotropic *L*-functions to describe the spatial arrangement of unmyelinated axon point patterns in nerve cross-sections, our method could be easily reimplemented utilizing pair correlation function (PCF) in place of the *L*-functions. PCF, which is related to *K* and *L*-functions, is of particular interest because it has previously been used in the context of biological microscopy (Sengupta et al., 2011, 2013; Veatch et al., 2012).

In addition *intensity* (or spatial density) and *interaction, regionality* is another aspect of the spatial organization that influences spatial descriptors and contributes to the concept of spatial similarity (or location). *Regionality* is the most intuitively understandable feature of spatial organization and denotes the absolute position of structures of interest within an object or region of interest (ROI). Regionality is not translation- and rotation-invariant, whereas interaction and intensity are (if not anisotropic). All of these characteristics are captured when spatial point patterns are processed using the tools described in this report.

### 2.3. Optimal transport framework and Sinkhorn distance

The *transport problem* distributes a certain amount of *mass* from a set of sources to a set of destinations at minimum cost. There are two major factors in a transport problem: the *cost function* and the *transportation plan*. The cost function defines a fixed, non-negative effort required to transport unit mass from a source to a destination. This cost may only depend on the distance between the source and the destination or on other additional factors; in the former case a Euclidean distance matrix between the sources and the destinations is a reasonable representation of effort.

Once the cost of transportation is represented, the remaining part of the problem involves transporting a non-negative amount of mass between sources and destinations, as described by a transportation plan. Various transportation plans result in different total costs, and the *optimal transport problem* (OT) aims to minimize this cost. The OT problem is *balanced* if the total mass at the sources equals the total mass at the destinations, and *unbalanced* otherwise (Peyré and Cuturi, 2019).

Let *r* and *c* be two *d* dimensional vectors representing the amount of mass at the *d* sources and the *d* destinations, respectively. The number of sources and destinations could differ, but they can be considered equal without loss of generality. Let *U*(*r, c*) be the set of all non-negative *d* × *d* matrices with row and column summing to *r* and *c*, respectively. Any matrix *P* ∈ *U*(*r, c*) describes a transportation plan that transports the mass in *r* to *c*. Given a *d* × *d* cost matrix *M*, the total cost of mapping *r* to *c* using the transportation plan *P* is $\underset{i,j}{\sum }P_{ij}M_{ij}$. Thus the OT problem between *r* and *c* given cost *M* can be formulated by Equation (5), where *D*_*M*_(*r, c*) is the optimal transport distance:


(5) $$\begin{array}{l} D_{M}(r,c)=\min_{P\in U(r,c)}\sum_{i,j}P_{ij}M_{ij},\\ \text{subject to }\sum_{j}P_{ij}=r_{i},\;\sum_{i}P_{ij}=c_{j},\;P_{ij}\geq 0,\;\forall i,j\leq d. \end{array}$$

The masses in *r* and *c* could be normalized to sum to one, and then both *r* and *c* can be interpreted as probability distributions.

For *D*_*M*_(*r, c*) to be a metric, the cost matrix *M* has to be a metric matrix (Avis, 1980; Brickell et al., 2008; Villani, 2009) satisfying the conditions shown in Equation (6).


(6) $$\begin{array}{l} \text{Non-negativity: }M_{ij}\geq 0,\\ \text{Identity: }M_{ii}=0,\\ \text{Symmetry: }M_{ij}=M_{ji},\\ \text{Triangle inequality: }M_{ij}\leq M_{ik}+M_{kj},\;\forall i,j,k\leq d. \end{array}$$

The OT is a convex optimization problem that can be solved using various approaches (Ahuja et al., 1993; Orlin, 1993). For a general cost matrix the computational cost scales as $O\left[d^{3}\text{log}\left(d\right)\right]$ (Pele and Werman, 2009), which prevents scaling the solution to large problem sizes. Earlier approximate solutions obtained by putting constraints on the cost matrix could result in a loss of applicability and performance (Grauman and Darrell, 2004). A later approximation to the original OT problem using an entropic regularization scheme was proposed by Cuturi (2013) to reduce the computational complexity. The scheme employs the Sinkhorn-Knopp matrix scaling algorithm (Sinkhorn and Knopp, 1967; Knight, 2008), and hence the name *Sinkhorn distance* for its objective function.

#### 2.3.1. Sinkhorn distance

A straightforward way of thinking about a transportation plan is by noticing that if a source contains more mass, it should originate more, and if a destination requires more mass, it should receive proportionally more. Such a transportation plan is represented by *rc*^*T*^, and the optimal plan *P* should be somewhere around the distribution *rc*^*T*^. Simply speaking, the idea of the entropic regularization scheme by Cuturi (2013) is to choose *P* from a smaller set near *rc*^*T*^, instead of the entire set *U*(*r, c*).

To capture these ideas, Cuturi (2013) imposes an additional constraint of Kullback-Leibler (KL) divergence on the OT formulation, as shown in Equation (7), and computes the Sinkhorn distance $D_{M,\alpha }^{*}\left(r,c\right)$. This constraint introduces a set *U*_α_(*r, c*) ⊂ *U*(*r, c*) from which an optimal transportation plan *P* is selected. The KL divergence distance between *P* and *rc*^*T*^ is set to be smaller than a predefined parameter α. In other words, *P* should belong to a distribution near *rc*^*T*^.


(7) $$\begin{array}{l} D_{M,\alpha }^{*}(r,c)=\min_{P\in U_{\alpha }(r,c)}\sum_{i,j}P_{ij}M_{ij},\\ \text{subject to }KL(P|rc^{T})\leq \alpha ,\;\sum_{j}P_{ij}=r_{i},\;\sum_{i}P_{ij}=c_{j},\;\forall i,j\leq d. \end{array}$$

The entropy (*h*) of the transportation plan (*P*) and the mass vectors (*r* and *c*) are given in Equation (8):


(8) $$\begin{array}{l} h(P)=-\sum_{ij}P_{ij}\log P_{ij},\\ h(r)=-\sum_{i}r_{i}\log r_{i},\;h(c)=-\sum_{j}c_{j}\log c_{j}. \end{array}$$

We proceed to express the KL divergence constraint in terms of the entropy:


(9) $$\begin{array}{l} KL(P|rc^{T})=\sum_{ij}P_{ij}\log\frac{P_{ij}}{r_{i}c_{j}}\\ =\sum_{ij}P_{ij}\log P_{ij}-\sum_{ij}P_{ij}\log r_{i}-\sum_{ij}P_{ij}\log c_{j}\\ =\sum_{ij}P_{ij}\log P_{ij}-\sum_{i}r_{i}\log r_{i}-\sum_{j}c_{j}\log c_{j}\\ \left[∵\sum_{j}P_{ij}=r_{i},\sum_{i}P_{ij}=c_{j}\right]\\ =-h(P)+h(r)+h(c)\leq \alpha . \end{array}$$

Thus the new constraint states that the entropy of *P* should be large enough to satisfy


h(P)≥h(r)+h(c)-α,

which constrains *P* to be chosen from the Kullback-Leibler ball of level α centered about *rc*^*T*^ (see Figure 1 in Cuturi, 2013).

This interpretation makes the OT problem non-convex, and an alternative formulation of Sinkhorn distance is required for ease of optimization. For every pair (*r, c*), each α corresponds to a Lagrange multiplier λ ∈ [0, ∞) such that $D_{M,\alpha }^{*}\left(r,c\right)=D_{M}^{\lambda }\left(r,c\right)$. The distance $D_{M}^{\lambda }$, shown in Equation (10), is called the dual-Sinkhorn divergence by Cuturi (2013).


(10) $$\begin{array}{l} D_{M}^{\lambda }(r,c)=\sum_{i,j}P_{ij}^{\lambda }M_{ij},\text{ where }P^{\lambda }=\underset{P\in U(r,c)}{\mathrm{argmin}}\sum_{i,j}P_{ij}M_{ij}-\lambda h(P),\\ \text{subject to }\sum_{j}P_{ij}=r_{i},\;\sum_{i}P_{ij}=c_{j},\;\forall i,j\leq d. \end{array}$$

By introducing two dual variables ϕ and ψ for each of the two equality constraints of Equation (10), the Lagrangian of the objective function can be written as Equation (11).


(11) $$\begin{array}{l} L(P,\phi ,\psi )=\sum_{i,j}P_{ij}M_{ij}-\lambda h(P)+\sum_{i}\phi_{i}(\sum_{j}P_{ij}-r_{i})\\ \;+\sum_{j}\psi_{j}(\sum_{i}P_{ij}-c_{j}). \end{array}$$

The derivative of the Lagrangian objective function with respect to *P*_*ij*_, for any pair (*i, j*), can be set to zero to obtain an extremum; the second derivative of the Lagrangian, ($\frac{\lambda }{P_{ij}}$), is positive since both the numerator and the denominator are positive, and thus we have obtained a minimizer of the Lagrangian.


(12) $$\begin{array}{l} \;\frac{\partial L}{\partial P_{ij}}=M_{ij}+\lambda +\lambda \log P_{ij}+\phi_{i}+\psi_{j}=0.\\ \Rightarrow \;P_{ij}=e^{-\frac{\phi_{i}}{\lambda }-\frac{1}{2}}.e^{-\frac{M_{ij}}{\lambda }}.e^{-\frac{\psi_{j}}{\lambda }-\frac{1}{2}}\\ \;\equiv u_{i}K_{ij}v_{j}\;[u_{i}=e^{-\frac{\phi_{i}}{\lambda }-\frac{1}{2}},v_{j}=e^{-\frac{\psi_{j}}{\lambda }-\frac{1}{2}},K=e^{-\frac{M}{\lambda }}] \end{array}$$

Given *K*, *r*, and *c*, the Sinkhorn-Knopp matrix scaling algorithm converges to a solution *P*^λ^ of the following form:


(13) $$\begin{array}{l} \exists u,v:P^{\lambda }=\text{diag}\left(u\right)K\text{diag}\left(v\right). \end{array}$$

*P*^λ^ should have the correct row and column sums, as shown in Equation (10). We deduce the update rule for the Sinkhorn-Knopp algorithms from those constraints in the following manner:


$$\begin{array}{l} \;\sum_{j}P_{ij}^{\lambda }=r_{i},\;\sum_{i}P_{ij}^{\lambda }=c_{j}\\ \Rightarrow \sum_{j}u_{i}K_{ij}v_{j}=r_{i}\;[\text{Equation 12}]\Rightarrow \sum_{i}u_{i}K_{ij}v_{j}=c_{j}\\ \text{ [Equation 12}]\\ \Rightarrow u_{i}\sum_{j}K_{ij}v_{j}=r_{i}\;\Rightarrow v_{j}\sum_{i}u_{i}K_{ij}=c_{j}\\ \Rightarrow u_{i}=r_{i}/\sum_{j}K_{ij}v_{j}\;\Rightarrow v_{j}=c_{j}/\sum_{i}u_{i}K_{ij} \end{array}$$

Thus the update rule for the Sinkhorn-Knopp algorithm can be written as Equation (14), where *v* can be initialized randomly.


(14) $$\begin{array}{l} u=r./(Kv),\\ v=c./(K^{T}u). \end{array}$$

Cuturi (2013) observes that the number of iterations in the Sinkhorn-Knopp algorithm is bounded independent of *d*. Thus, the cost of computing $D_{M}^{\lambda }$ is $O\left(d^{2}\right)$, which is an improvement over $O\left[d^{3}\text{log}\left(d\right)\right]$. Cuturi (2013) describes an approach to compute the Sinkhorn distance $D_{M,\alpha }^{*}\left(r,c\right)$ through the dual-Sinkhorn divergence $D_{M}^{\lambda }\left(r,c\right)$, and also reports that the dual-Sinkhorn divergence does not perform worse than the classic optimal transport distances. Therefore, we use the dual-Sinkhorn divergence to measure the distance between the spatial statistics of the point patterns in our experiments and refer to as the Sinkhorn distance. We utilize the R packages *T4transport* (You, 2022) and *Barycenter* (Klatt, 2018) for computing the dual-Sinkhorn divergences, and *spatstat* (Baddeley et al., 2015) for spatial point pattern analysis.

## 3. Experiments and results

We represent the unmyelinated axonal arrangements in the vagus and pelvic nerve cross-sections as spatial point patterns. We intend to quantify (using the Sinkhorn distance) similarities between the point patterns in terms of the following spatial features:

1. spatial intensity,
2. local inhomogeneous *L*-function,
3. local inhomogeneous anisotropic *L*-function with
  (a) horizontal and
  (b) vertical sectors.

For the horizontal and vertical cases, we choose sectors forming 15° segment around the horizontal (0°) and vertical (90°) axes, respectively. We attach the above-mentioned spatial features to the point patterns as marks (described in Section 2.2). We compute the Sinkhorn distance between every pair of point patterns, for the four spatial features, in two different manners:

1. using the spatial point patterns directly (in Section 3.4) and
2. using the map of the spatial features constructed by kernel-smoothing (in Section 3.5).

The Sinkhorn distance between every pair of nerve cross-sections is then used to construct a symmetric Sinkhorn distance matrix and visualized in an embedded space *via* multi-dimensional scaling. In the following three subsections, we describe a few preprocessing and parameter selection tasks required for configuring the spatial features, before going into the experimental details.

### 3.1. Interaction distance configuration

A critical issue regarding the computation of local inhomogeneous *L*-functions is determining the interaction distance (*r*), described in Section 2.2. The point patterns constructed from the nerve cross-sections differ in size, as do their interaction ranges. The preferred choice for the interaction distance is the one that can reasonably separate the spatial features of interest present in the point patterns. We compute a range of interactions that are common for all the point patterns and configure the interaction distance using two approaches: (a) based on the standard deviations of the inhomogeneous *L*-function of all the point patterns and (b) based on the F-ratio (analysis of variance) of the inhomogeneous *L*-function of the point patterns grouped as vagus vs. pelvic, within the expected range. Other strategies for choosing *r* or a linear combination of multiple *r* values are also possible (See Section 3.4.1).

### 3.2. Translation and rotation normalization

The optimal transport distance is not invariant under translations and rotations (Wang et al., 2013). This is critical in the case of analyzing the point patterns because spatial inhomogeneity and anisotropy depend largely on the placement and orientation of the point patterns. To provide translation invariance, we scale the point patterns maintaining proportionality, align the center of mass to the origin, and apply the necessary 0-padding around the biological structures.

Ensuring rotation invariance is non-trivial. In an ideal setting, the information regarding the orientation of the biological structures would be available directly to the analyst. Unfortunately, the experimental and instrumental setting may not always allow the orientation of the samples to be maintained during the specimen preparation and the imaging process. Therefore, we implemented a post-hoc minimization process as a workaround. While computing the Sinkhorn distance between the spatial intensities of a pair of point patterns, we keep the orientation of one of them unchanged and rotate the other one about the origin by multiple θ values (θ= 45° in our experiments). We compute the Sinkhorn distance for all possible values of θ and keep the orientation that provides the smallest Sinkhorn distance result. We use this identified orientation for the computation of other spatial features. This method provides a reproducible procedure in the absence of known anatomical orientation data.

### 3.3. The entropic regularization parameter

We refer to the coefficient of the entropy of the transportation plan *h*(*P*) in the dual-Sinkhorn divergence formulation shown in Equation (10), λ, as the entropic regularization parameter. As λ → 0, Sinkhorn distance approaches the optimal transport distance (Wasserstein distance, provided that the cost is Euclidean distance). As λ increases, the computation results in different approximations of the optimal transport distance, i.e., the Sinkhorn distances. Tuning the appropriate entropic regularization parameter is an important task. We can consider two scenarios: (a) selecting an entropic regularization parameter that provides better separation between analyzed instances and (b) selecting an entropic regularization parameter that makes the Sinkhorn distance a more accurate approximation of the exact optimal transport distance. Thus, parameter tuning is a trade-off between favoring the utility of the method (and lower computational cost) and the accuracy of the approximation.

Smaller values of the λ parameter produce Sinkhorn distances that more closely approximate Wasserstein distances (Cuturi, 2013). Therefore, if computational resources are not limited, smaller λ values are preferable to larger values. To estimate the computational cost, we tried λ = 0.01, 0.05, 0.1, 0.5, 1.0, 2.0, 5.0, but we settled on λ = 0.01.

### 3.4. Sinkhorn distance between spatial point patterns

In this section, we compute Sinkhorn distance between the spatial point patterns (directly) of the nerve cross-sections, for the four spatial features mentioned earlier. Let *S*_1_ and *S*_2_ be two spatial point patterns, with *n*_1_ and *n*_2_ number of points respectively. Considering an optimal transport problem between *S*_1_ and *S*_2_, we assume that each point in *S*_1_ contains $\frac{1}{n_{1}}$ amount of mass (*r*), therefore the total mass $=\frac{1}{n_{1}}\times n_{1}=1$. Similarly, each point in *S*_2_ requires $\frac{1}{n_{2}}$ amount of mass (*c*), so the total mass $=\frac{1}{n_{2}}\times n_{2}=1$. Thus the problem is to transport the mass from *S*_1_ to *S*_2_ (balanced). The inputs for the computations are the spatial locations of the points in *S*_1_ and *S*_2_. Therefore, we can compute the Euclidean distance matrix between them to be the cost matrix *M*, of dimension *n*_1_ × *n*_2_. Here, the cost matrix *M* captures the spatial intensity of the point patterns. Further, we compute the transportation plan *P*, of dimension *n*_1_ × *n*_2_, using the formulation described in Section 2.3.1 and obtain the Sinkhorn distance, which provides a measure of similarity of the spatial intensity between *S*_1_ and *S*_2_.

In the cases of the three other spatial features, the cost matrix (*M*) remains the same (the spatial location of the points are unchanged), but the amount of mass produced (*r*) or required (*c*) at each point changes. The local inhomogeneous *L*-function attaches a numerical value to each point in a point pattern that captures its local spatial interaction within a certain interaction distance. Instead of a uniform mass amount, we may assume that each point in *S*_1_ and *S*_2_ is assigned an amount of mass that equals its local inhomogeneous *L*-function value. We normalize the values assigned to each point pattern to sum to one. Then we can compute the transportation plan *P* in the same manner as described above. The resultant Sinkhorn distance gives us a measure of similarity of the local spatial interaction between *S*_1_ and *S*_2_. The Sinkhorn distances for the anisotropic spatial features, the local inhomogeneous anisotropic *L*-function with horizontal and vertical sectors, are also computed similarly.

We have 29 nerve cross-sections in the dataset and once we compute the Sinkhorn distance between every pair, we can construct Sinkhorn distance matrices of dimension 29 × 29, for each of the four spatial features. We use multi-dimensional scaling to embed the Sinkhorn distance matrices in 2-D to illustrate the computed Sinkhorn distance between the spatial features and interpret the notion of similarity (or dissimilarity) between the nerve cross-sections. We denote the new embedded 2-D space as the *Sinkhorn space*.

The application of the optimal transport (OT) solution to define the similarity of spatial point patterns is dependent on all of the spatial organization characteristics described in Section 2.2. Intensity differences are conveyed by the number of points at a given location relative to other sites where mass for transportation is present. As point weights in OT, the values of the local (inhomogeneous and anisotropic) *L*-functions represent information about point-point interactions. Lastly, the movement of mass from one area in ROI to another directly captures the sense of regionality. Although it is possible to simulate pattern arrangements that emphasize only one aspect of spatial organization while diminishing the influence of the others, this will not lead to the development of an intuitive sense of computed distance in a real-world setting. As with numerous other mathematical concepts (and distance in particular), the interplay between the various aspects of spatial organization manifested in biological samples defies simple models.

The architecture of the unmyelinated axons in the vagus and pelvic nerve cross-sections are examples of spatial complexity arising from the combination of multiple aspects of organization. Therefore, it might be difficult to develop an intuitive sense of the Sinkhorn distance between the axonal organization of these structures and their placement in the Sinkhorn space. Before discussing biological point patterns, we present a number of simulated point patterns to illustrate only a few particular scenarios emerging as realizations of pre-defined spatial point-pattern processes (they do not represent the entire landscape of possible phenotypic manifestations).

#### 3.4.1. Sinkhorn distance for simulated point patterns

**Figure 7:**
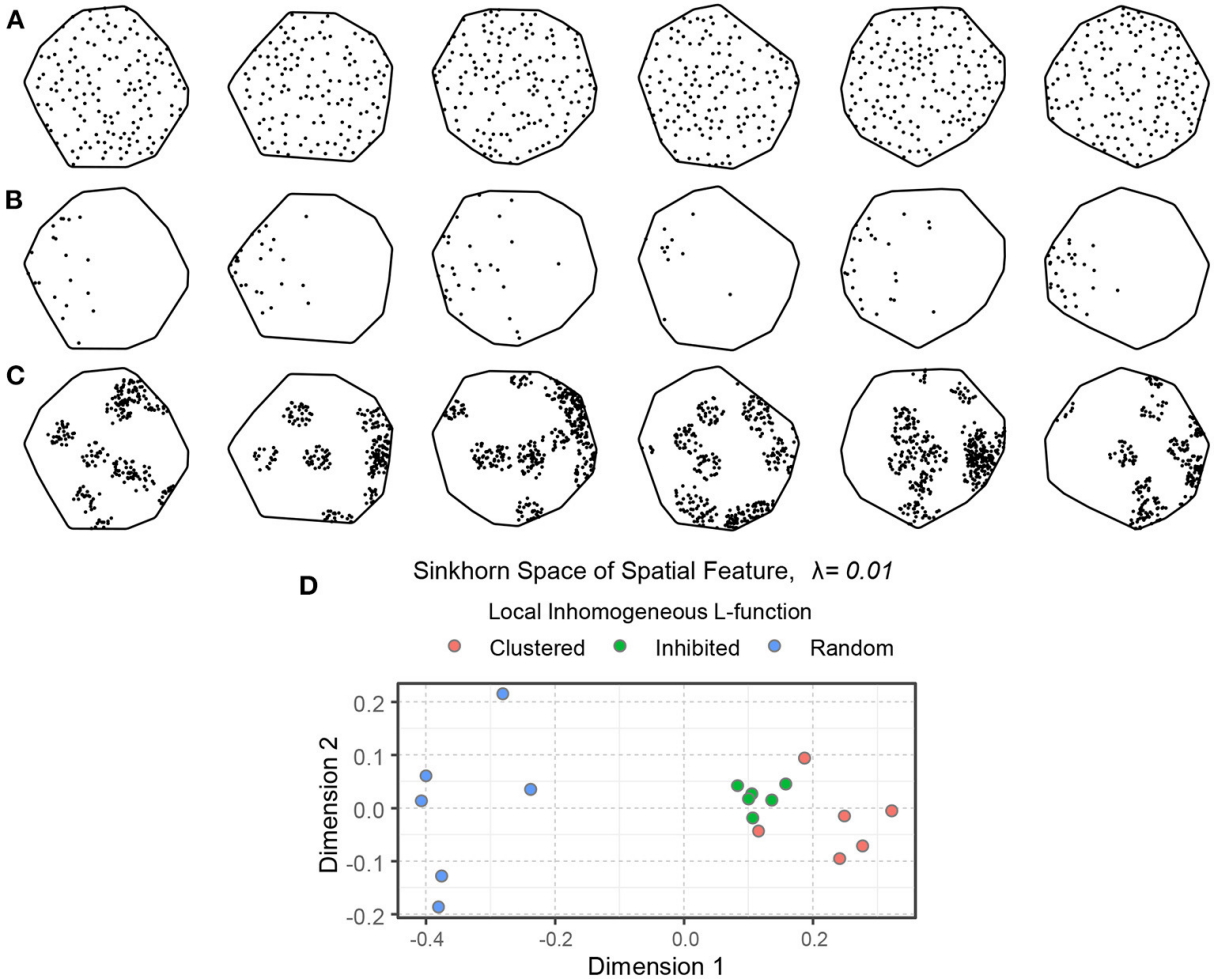
An illustration of spatial point patterns with different inhomogeneous spatial interactions. **(A)** Spatial inhibition. **(B)** Spatial randomness. **(C)** Spatial clustering. The intensity functions of the random and clustered patterns introduce some anisotropy. **(D)** An embedding of the point patterns in the Sinkhorn space. The inhibited, random, and clustered point patterns are shown in green, blue, and orange, respectively.

Figures 7A–C show 18 simulated spatial point patterns with different spatial *interactions*—inhibition, randomness, and clustering. These point patterns were formed by inhomogeneous point processes, namely the hardcore process, the Poisson process, and the Matern cluster process (Baddeley et al., 2015). For the simulation, we used a hardcore process with 400 points per unit area and a hardcore distance of 0.04 (the points are not allowed to be within 0.04 unit of distance from each other, ensuring inhibition). We used a Poisson process with inhomogeneous intensity function ρ_*r*_(*x, y*), shown in Equation (15), and a Matern cluster process with inhomogeneous intensity function ρ_*c*_(*x, y*) for the cluster centers, shown in Equation (16), with cluster radius 0.10 and 25 points per cluster. The intensity functions ρ_*r*_(*x, y*) and ρ_*c*_(*x, y*) introduce some anisotropy in the corresponding point patterns. The embedding of the local inhomogeneous *L*-function of the simulated point patterns in the Sinkhorn space is depicted in Figure 7D, where point patterns with similar spatial interaction are mapped close to each other and a clear separation can be seen between samples with different spatial interaction.


(15) $$\begin{array}{l} \rho_{r}\left(x,y\right)=100\times \exp\left(-5x\right) \end{array}$$

(16) $$\begin{array}{l} \rho_{c}\left(x,y\right)=10\times \exp\left(2|x|-1\right) \end{array}$$

Similar simulations can be used for any type of spatial point pattern, providing an explainable, semi-mechanistic rationale for the emergence of the patterns and an interpretable representation of their properties and reasons for separation.

**Figure 8:**
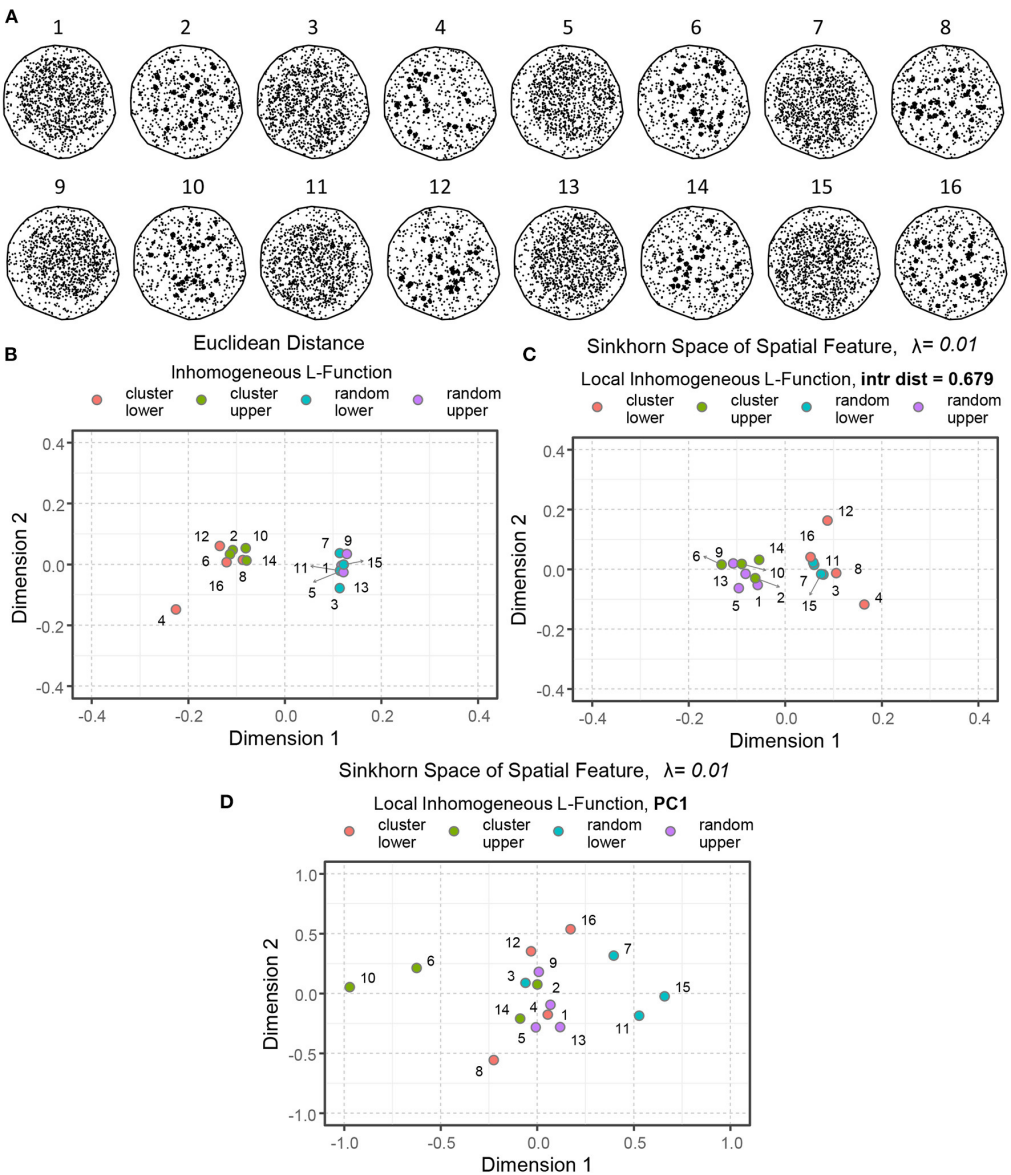
An illustration of spatial point patterns with different interactions and regionality. **(A)** Simulated examples of spatial point patterns: concentrated in the upper right (1, 2, 5, 6, 9, 10, 13, 14) and the lower left (3, 4, 7, 8, 11, 12, 15, 16) corners demonstrate regionality. The points are organized randomly in the odd-numbered examples and clustered in the even-numbered instances. **(B)** Euclidean distance between the inhomogeneous *L*-functions of the simulated models. **(C)** Sinkhorn distance between the local inhomogeneous *L*-functions of the simulated examples at a large interaction distance (*r* = 0.679). **(D)** Sinkhorn distance between the first principal component (PC) of local inhomogeneous *L*-functions of the simulated examples over a set of interaction distances. The point patterns of different interactions and regionality are shown in different colors.

Figure 8A depicts a set of spatial point patterns with different *interactions* and *regionality*. The points are concentrated in the upper right (examples 1, 2, 5, 6, 9, 10, 13, and 14) and the lower left (examples 3, 4, 7, 8, 11, 12, 15, and 16) corners to demonstrate difference in regionality. The points are organized randomly in the odd-numbered examples, and clustered in the even-numbered examples, within their corresponding regions. Figures 8B–D illustrate the notion of similarity capturing *interaction* and *regionality* as the two key aspects of spatial organization.

When averaged for all the points, the second-order spatial statistics, such as the inhomogeneous *L*-functions, describe the overall spatial interaction of a point pattern (as shown in Figures 4B, 5) but lose the ability to capture the notion of regionality. Therefore, by computing pairwise Euclidean distances between the simulated examples' inhomogenous *L*-functions and embedding the results in the Euclidean space, we communicate only the distance between point-pattern interactions while excluding the regionality contribution entirely. See Figure 8B, where the random patterns are primarily present on the right side of the plot, whereas the clustered patterns lie on the left.

Figure 8C illustrates another valid concept of similarity but constructed with a different emphasis. In this example, the local inhomogeneous *L*-functions were computed with a very large interaction distance. Therefore, the effect of regionality dominates. The resulting Sinkhorn embedding shows patterns with points concentrated on one side of ROI separating from the patterns in which points were concentrated on the other side of ROIs. Changing the interaction distance *r* at which the local *L*-functions are computed provides flexibility on how much influence of regionality is incorporated into the statistics and, correspondingly, to which extent the notion of similarity is shaped by regionality vs. point-point interactions.

Alternatively, one can employ singular value decomposition to compress all the information regarding interactions at various *r* values. In this case, the Sikhorn distance would operate on the linear combination of *r* distances that convey the largest variance. This case is illustrated in Figure 8D, where both aspects of the spatial organization are employed to compute point-pattern similarity. Consequently, the visualized distances between point patterns are influenced by the interaction and the regional organization. The degree to which different aspects of spatial architecture and interaction distance should shape the metric is a choice that must be made based on domain knowledge regarding the anatomy and significance of varying levels of structural organization. Finally, it is worth mentioning that the Sinkhorn distance between two point patterns with similar interaction but different regionality might be approximately equal to the Sinkhorn distance between two point patterns with vastly different interaction characteristics but similar regionality.

#### 3.4.2. Sinkhorn distance for biological point patterns

**Figure 9:**
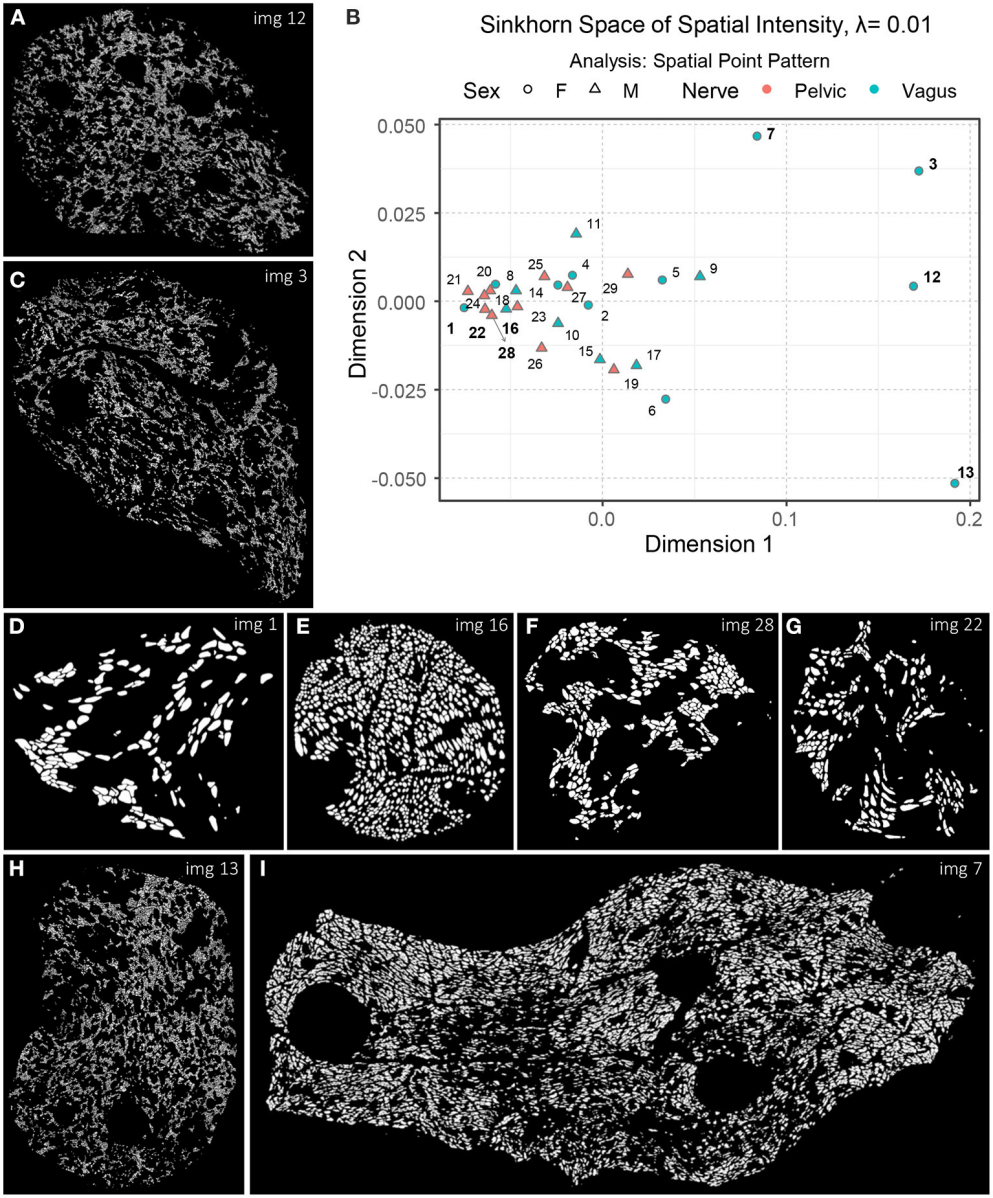
**(A, C–I)** A set of images of the segmented unmyelinated axons in the nerve cross-sections, labeled with the Image ID. **(B)** An embedding of the spatial intensity of the spatial point patterns in the Sinkhorn space for entropic regularization parameter λ = 0.01. The vagus and the pelvic samples are shown in cyan and orange [circles for female (F) and triangles for male (M)], respectively, and labeled with the Image ID listed in Table 1.

Now that we have an idea of how different aspects of the spatial organization can be captured with Sinkhorn distance and visualized in the Sinkhorn space, we describe the experimental results of the biological point patterns. Figure 9B shows an embedding of the spatial intensity of the spatial point patterns (used directly) of the nerve cross-sections in the Sinkhorn space for entropic regularization parameter λ = 0.01. The vagus and the pelvic samples are shown in cyan and orange, respectively, and labeled with the Image ID listed in Table 1. Figure 9H shows Image 13 (vagus), which is positioned far from the other samples in the Sinkhorn space. It is the largest sample in our dataset regarding image size and the number of segmented unmyelinated axons. It is also the only sample from a left cervical trunk. Figures 9A, C display Image 12 and Image 3 (both vagus) respectively. They are collected from the right cervical trunks. Figure 9I shows Image 7 (vagus) from abdominal vagus posterior trunk. Considering the spatial intensity, these four vagus samples are embedded far apart and are visually different. The rest of the samples are positioned in proximity, yet we can see a rightward tendency in the vagus samples than the pelvic ones. Figures 9D, E show Image 1 and Image 16 (both vagus), respectively. They look spatially different from the vagus samples discussed so far and are embedded at the leftmost part of the Sinkhorn space, far from those samples. However, they have a similar spatial organization as Image 28 and Image 22 (both pelvic) shown in Figures 9F, G and are embedded closer in the Sinkhorn space.

Although one can intuitively understand the global differences in spatial intensity of the point patterns by looking at the images of segmented unmyelinated axons in the nerve cross-sections, our approach can quantify and visualize the differences with the Sinkhorn distance between every pair of samples, resulting in a map of patterns. For instance, in Figure 9B, the Sinkhorn distances between the spatial intensity of Image 1, and Image 3 and Image 12 are 0.257 and 0.254, respectively, whereas the distance between Image 3 and Image 12 is 0.107. Again, Image 16 and Image 28 have respectively distances 0.054 and 0.048 from Image 1.

**Figure 10:**
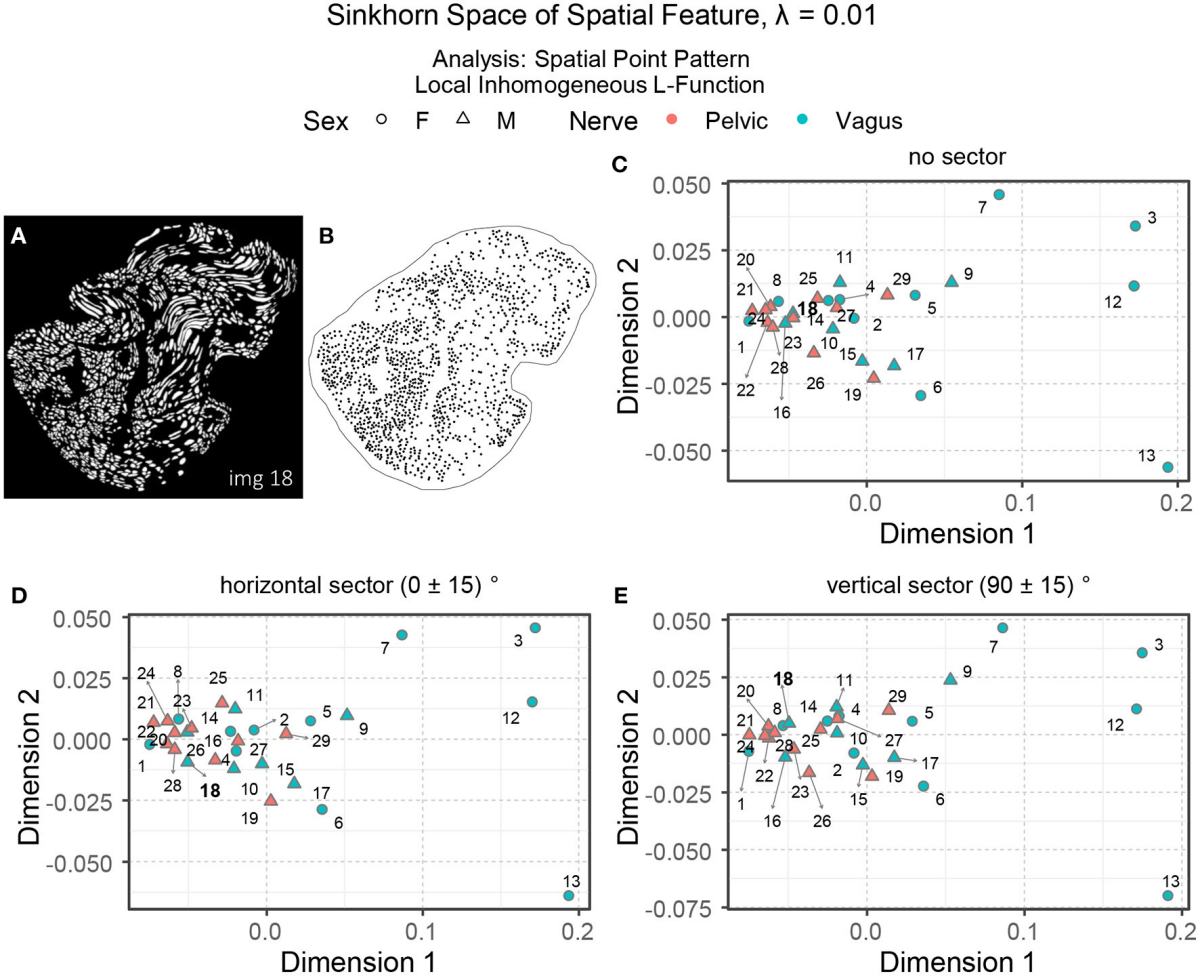
**(A, B)** The segmented unmyelinated axons and the spatial point pattern of Image 18 (vagus) listed in Table 1. **(C–E)** The embeddings of the local inhomogeneous and anisotropic *L*-functions (no sector, horizontal sector, and vertical sector) of the spatial point patterns in the Sinkhorn space for entropic regularization parameter λ = 0.01. The vagus and the pelvic samples are shown in cyan and orange [circles for female (F) and triangles for male (M)], respectively, and labeled with the Image ID listed in Table 1.

Interpreting spatial statistics, such as local inhomogeneous and anisotropic *L*-functions, can be more challenging than understanding raw spatial intensity. Figures 10C–E show three embeddings of the spatial features in the Sinkhorn space for λ = 0.01: the local inhomogeneous *L*-function, the local inhomogeneous *L*-function with horizontal sector and vertical sector, respectively. With a few exceptions, the overall landscape in the embeddings is similar to the one for the spatial intensity shown in Figure 9B. Figure 10A showing the segmented unmyelinated axons for Image 18 (vagus) contains several elongated axons. The elongated axons make the spatial arrangement of centroids in the corresponding point pattern (see Figure 10B) quite sparse and direction-oriented (anisotropic) in certain regions. The different positioning of Image 18 in the embeddings can reflect these characteristics.

### 3.5. Sinkhorn distance between maps of spatial features

Here, we compute the Sinkhorn distance between every pair of point patterns using the map of the spatial features constructed by kernel-smoothing. When we consider spatial point patterns directly (as in Section 3.4), the mass (corresponding to the spatial intensity or any other spatial feature attached as marks) to be transported is concentrated at the exact location of a point. As we apply kernel-smoothing to the point pattern, the concentrated mass at any point diffuses into its neighborhood. This step can help capture the notion of regionality in the kernel-smoothed maps while computing Sinkhorn distances. Notably, the kernel-smoothing reduces the influence of the differing number of points in the compared point patterns on the resulting Sinkhorn distances. The transportation-based metrics are well-suited for quantifying differences between bitmaps in which pixel values can be interpreted as transportable mass without strict geometric constraints (Rubner et al., 2000; Grauman and Darrell, 2004; Haker et al., 2004; Chefd'Hotel and Bousquet, 2007; Wang et al., 2011, 2013).

**Figure 11:**
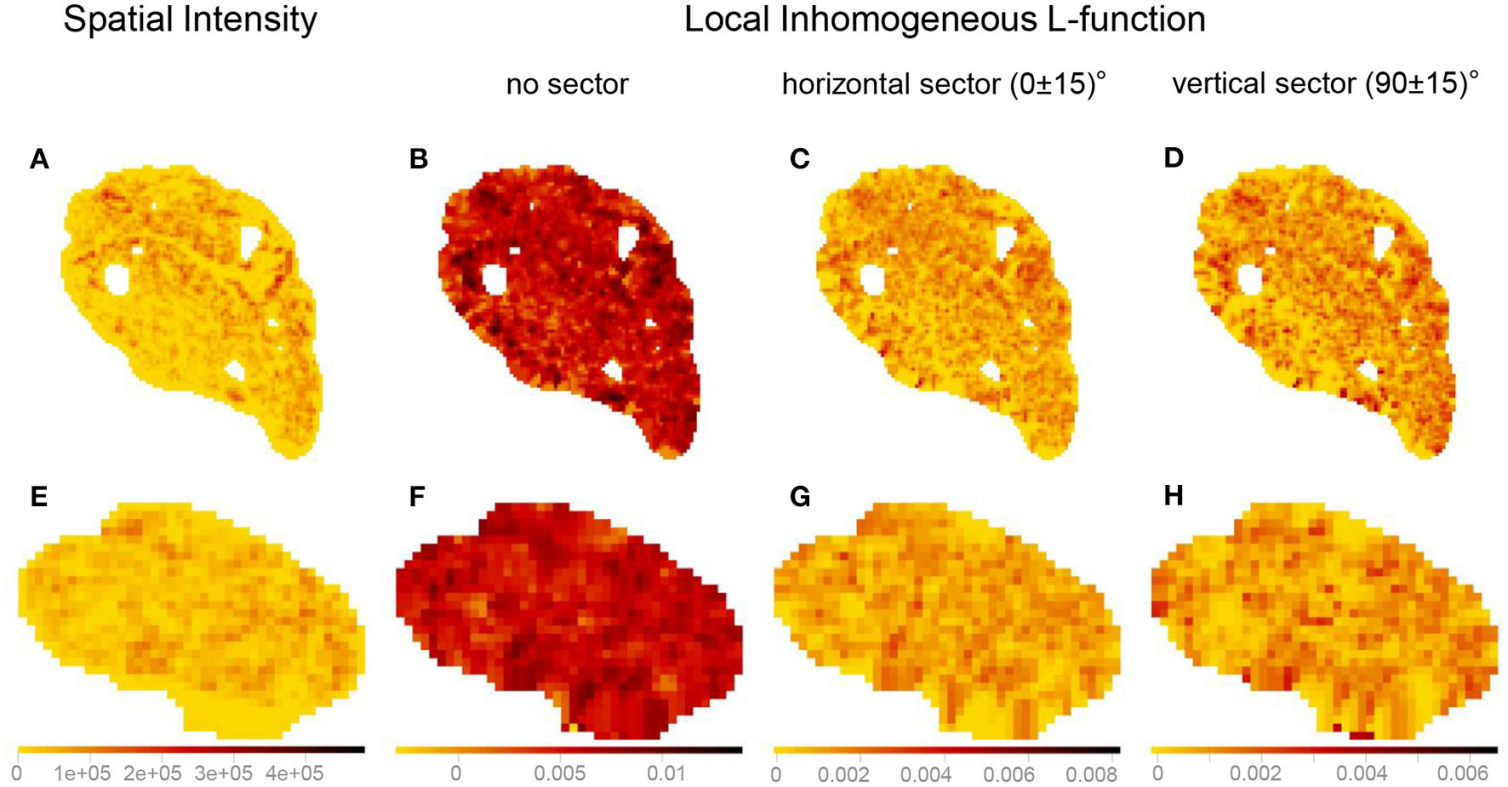
Visualizing the kernel-smoothed spatial features of Image 3 (vagus) and Image 29 (pelvic) listed in Table 1. **(A, E)** Spatial intensity. **(B, F)** Local inhomogeneous *L*-function. **(C, G)** Local inhomogeneous *L*-function with the horizontal sector. **(D, H)** Local inhomogeneous *L*-function with the vertical sector. The scale bars show the range of values for each spatial feature separately (column-wise). The kernel-smoothed bitmaps were downsampled for reasonable runtime and memory requirements.

The kernel-smoothed spatial intensities, as well as marks attached to a point pattern, can be depicted as bitmaps, where the pixel values represent kernel-smoothed intensity values or other quantities derived from the marks (e.g., local inhomogeneous and anisotropic *K*- and *L*-functions). The kernel-smoothed spatial features of Images 3 (vagus) and 29 (pelvic) listed in Table 1 are shown in Figure 11. The bitmaps for the local inhomogeneous *L*-function, demonstrated in Figures 11B, F, have higher values of the spatial feature compared to their anisotropic counterparts shown in Figures 11C, D, G, H and slight shifts in values at certain locations are observed between the bitmaps of the horizontal and vertical sectors. Quantifying similarities between the kernel-smoothed bitmaps can be performed using Sinkhorn distance just like quantifying similarities between the spatial features of the original point patterns.

Let *I*_1_ and *I*_2_ be the centered (0-padded as necessary) kernel-smoothed maps of the spatial intensity of the point patterns *S*_1_ and *S*_2_, respectively. The pixel values in *I*_1_ and *I*_2_ are normalized to sum to one, and the value at each pixel is considered the amount of mass contained (*r*) or required (*c*) at that pixel. The location of the pixels is not known beforehand, so we construct a unique grid [0, 1]^2^ over which the pixel locations of *I*_1_ and *I*_2_ are defined. The cost matrix *M* is the Euclidean distance matrix computed from the [0, 1]^2^ grid. The transportation plan *P* and the Sinkhorn distance between *I*_1_ and *I*_2_ are computed in the previously described manner. The Sinkhorn distances between the maps representing the other three spatial features are also calculated in the same fashion. Constructing the Sinkhorn distance matrix and visualizing embeddings in the Sinkhorn space are also done in the same way as described in Section 3.4.

**Figure 12:**
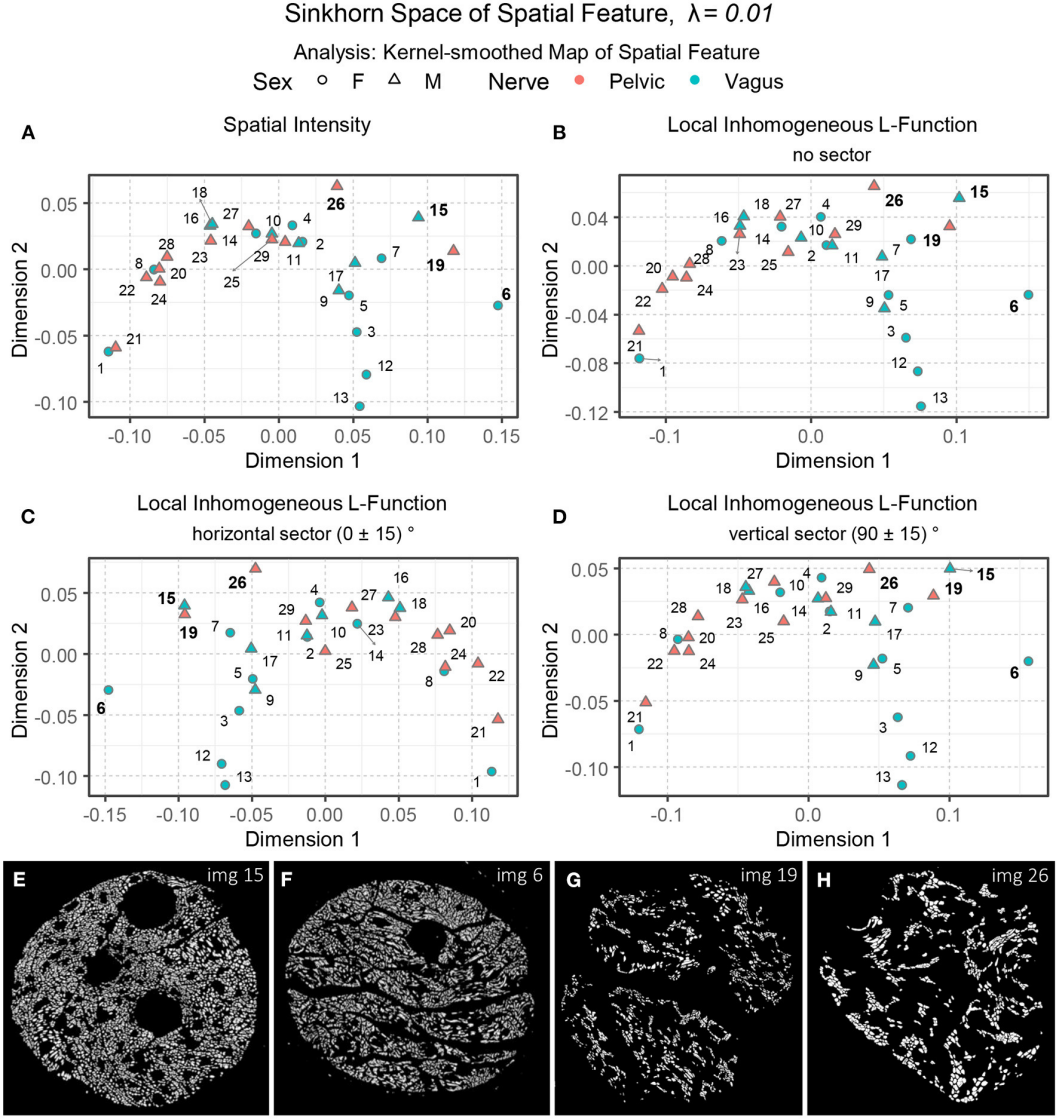
**(A–D)** The embeddings of the kernel-smoothed spatial intensity and the local inhomogeneous and anisotropic *L*-functions (no sector, horizontal sector, and vertical sector) of the spatial point patterns in the Sinkhorn space for entropic regularization parameter λ = 0.01. The vagus and the pelvic samples are shown in cyan and orange [circles for female (F) and triangles for male (M)], respectively, and labeled with the Image ID listed in Table 1. **(E–H)** The segmented unmyelinated axons of Image 15 and 6 (vagus) and Image 19 and 26 (pelvic) listed in Table 1.

Figure 12A shows an embedding of the kernel-smoothed maps of the spatial intensity of the point patterns in the Sinkhorn space of λ = 0.01. The vagus and the pelvic samples are shown in cyan and orange, respectively, and labeled with the Image ID listed in Table 1. The vagus samples 3, 7, 12, and 13 are embedded at a distance from the rest of the samples, and this trend was also observed in Figure 9B, when we processed the point patterns directly. However, vagus samples 6 and 15, and pelvic samples 19 and 26 (see Figures 12E–H), which were positioned close to the rest of the samples in Figure 9B, are located far apart in the right-most region of the embedding in Figure 12A. Therefore, some characteristics of the spatial intensities that were not captured during the processing of the raw point patterns became apparent when spatial feature maps were employed. The embeddings of kernel-smoothed local inhomogeneous and anisotropic *L*-function are illustrated in Figures 12B–D, portraying a similar trend overall, where the perceptually comparable vagus and pelvic samples are positioned in proximity, the rest are far apart, and the vagus samples are more spread out.

### 3.6. Insights regarding the spatial architecture

We computed the Sinkhorn distances between every pair of nerve cross-sections for the four spatial features using spatial point patterns directly (data shown in Section 3.4) and kernel-smoothed bitmaps representation of the spatial features (data shown in Section 3.5). The resulting Sinkhorn embeddings are displayed in Figures 9, 10, 12. The created Sinkhorn space allows us to observe the similarities (or dissimilarities) of spatial intensities and second-order spatial properties.

The secondary statistical analysis performed on the embedded patterns generated by kernel-smoothing to mitigate the effects of the unequal number of axons revealed that the difference in spatial architecture between the vagus and pelvic nerves is relatively small (Mahalanobis distance Δ = 0.91). However, the sample size is insufficient to determine whether this observed difference reflects biological reality or results from random chance. With *n*_pelvic_ = 11 and *n*_vagus_ = 18, the achieved power (1-β) is only 0.6. In order to confirm the spatial architectural difference between vagus and pelvic nerve cross-sections, the required data set size should be at least *n* = 26 per class for 1-β = 0.8 and α = 0.05 in 2-D embedding, according to the collected preliminary results. In other words, any future research on the potential architectural difference between peripheral nerves' axonal organization (or modulation of a such organization due to pathology or treatment) must use these preliminary effect sizes as a reasonable basis for necessary power analysis needed for experimental design.

On the other hand, there is a substantial difference between the nerve cross-sections of males and females (Δ=1.246, Hotelling T^2^ test *p*-value = 0.013). However, this effect must be confirmed and replicated with an unconfounded sample set in which the correlation between sex and cross-section origin (pelvic vs. vagus) is absent. The result reported here is based on the assumption that there is indeed no statistically significant difference between pelvic and vagus.

**Figure 13:**
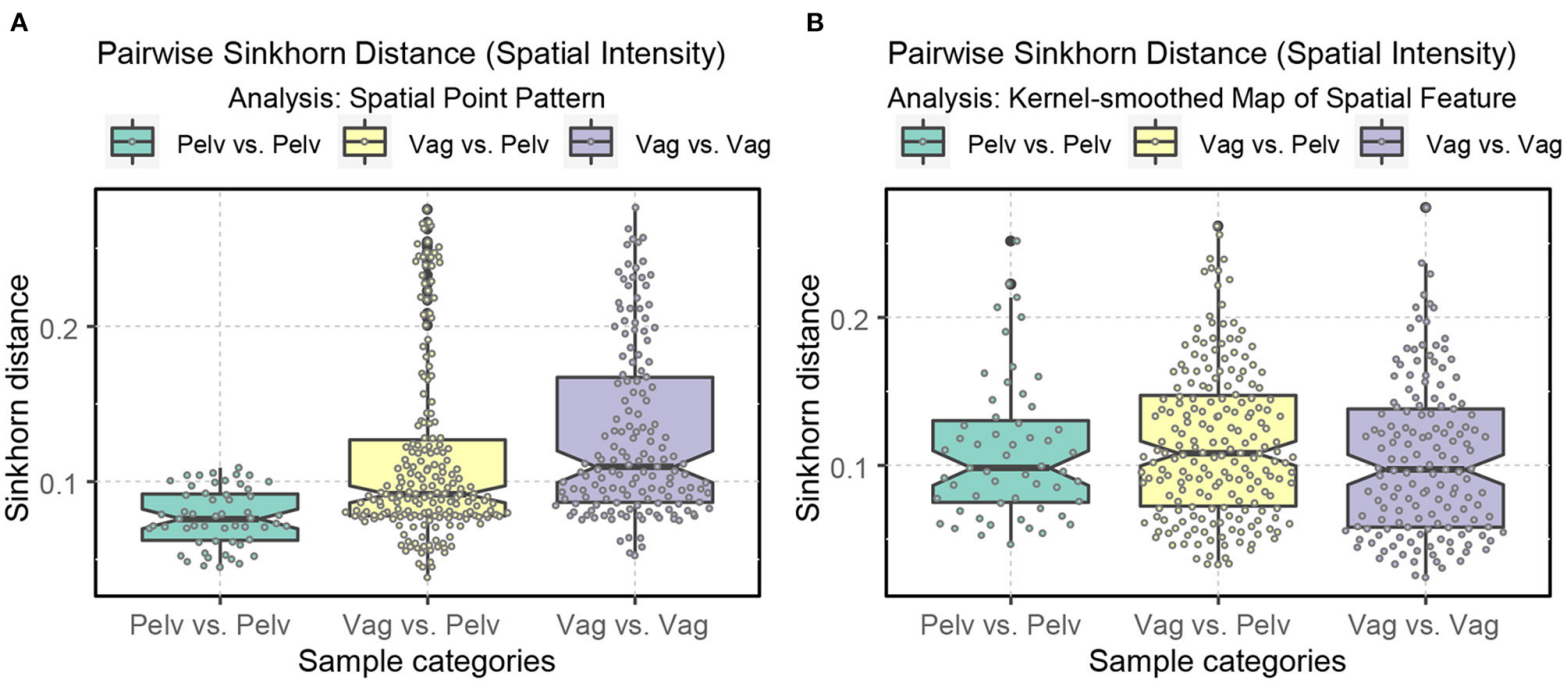
Boxplots displaying the ranges of Sinkhorn distance between the spatial intensity of the nerve cross-sections. Pelv: pelvic; Vag: vagus. **(A)** Analysis of the spatial point patterns directly. **(B)** Analysis of the kernel-smoothed maps of the spatial features. The points show the individual Sinkhorn distances, revealing the hidden distribution.

Regarding intraclass variability, vagus samples exhibit a significantly greater diversity of spatial architecture than pelvic samples when the raw spatial patterns are directly compared (Figure 9B). However, this difference disappears when the kernel-smoothed spatial patterns are compared, indicating that it was driven mainly by the difference in the number of axons rather than the spatial architecture (Figures 12, 13).

**Figure 14:**
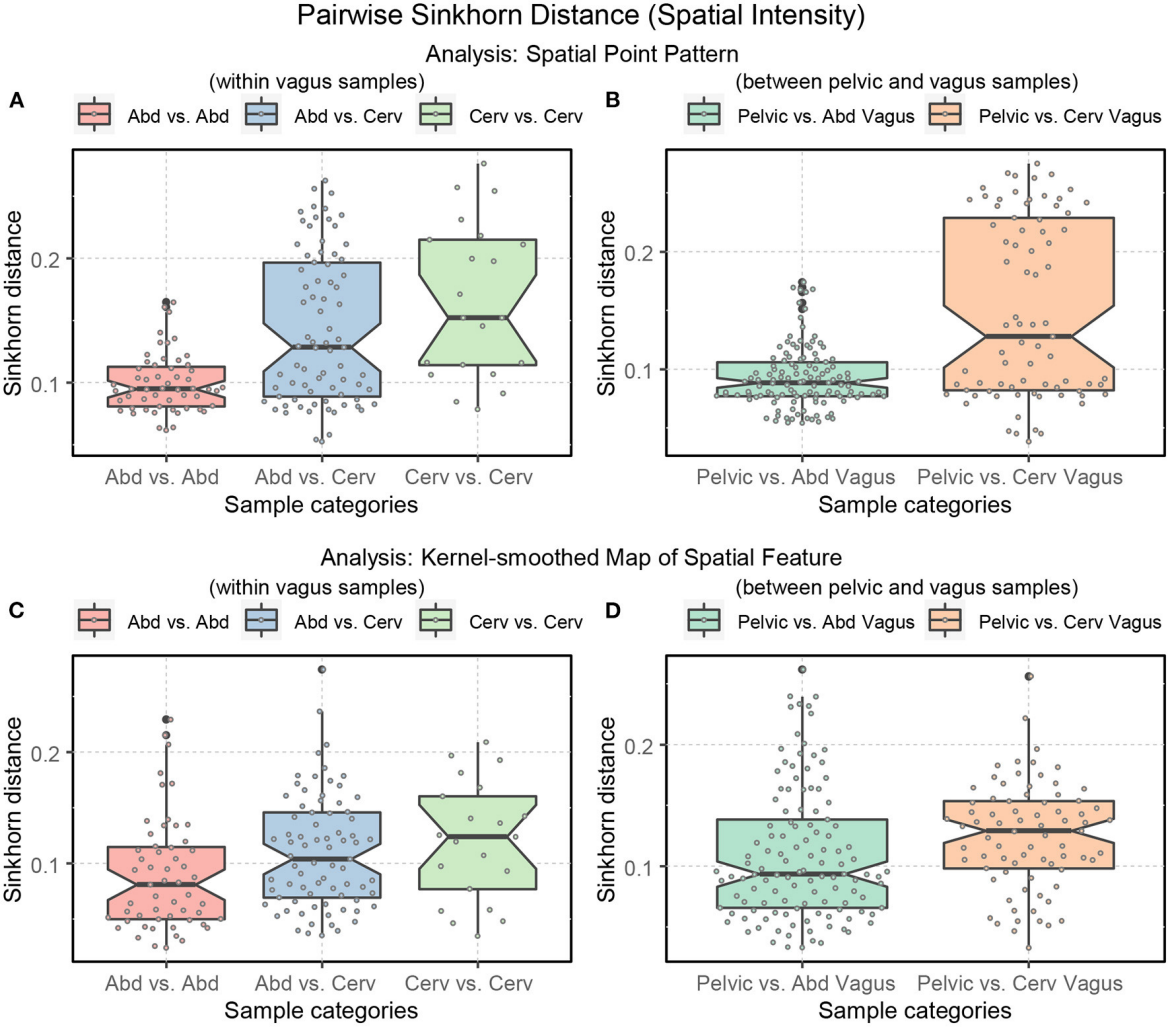
Boxplots displaying the ranges of Sinkhorn distance between the spatial intensity of the sub-categories of the nerve cross-sections. Abd: abdominal vagus; Cerv: cervical vagus, and pelvic. **(A, B)** Analysis of the spatial point patterns directly. **(C, D)** Analysis of the kernel-smoothed maps of the spatial features. The points show the individual Sinkhorn distances, revealing the hidden distribution.

The vagus samples in our dataset are collected from the abdominal and cervical regions (see Table 1). Thus, looking into the degree of variability of the Sinkhorn distance within the sub-categories of the vagus samples and between the pelvic samples is helpful. Figure 14 illustrates the range of Sinkhorn distance between the spatial intensity of every pair of nerve cross-sections (for both types of analysis), categorized as the following:

1. within vagus samples
  (a) intra-class measurements (i) (abdominal vagus vs. abdominal vagus)
  (b) intra-class measurements (ii) (cervical vagus vs. cervical vagus)
  (c) inter-class measurements (abdominal vagus vs. cervical vagus)
2. between pelvic and vagus samples
  (a) inter-class measurements (i) (pelvic vs. abdominal vagus)
  (b) inter-class measurements (iii) (pelvic vs. cervical vagus)

In Figure 14, we see the ranges of the Sinkhorn distances for the abovementioned sub-categories. The degree of variability is more prominent in the analysis of the raw spatial point patterns (Figures 14A, B) compared to the analysis of the kernel-smoothed bitmaps representing spatial features (Figures 14C, D). This observation applies not just to spatial intensity but also statistical second-order spatial statistics. Figure 14 shows that variability within the abdominal vagus samples is substantially lower than the spatial variability within the cervical vagus cross-sections (*p*-value < 0.001). However, this notion has not been confirmed when looking at Figure 14C, which is based on pre-processing that eliminates the effect of axons' number. As before, this is most likely due to the low statistical power of the available sample size. The number of abdominal (55) and cervical (21) pairwise measurements is too small to confidently demonstrate the observed standardized effect size of Δ = 0.59. The required number of measurements for such effect size should be at least 45 per class to achieve 1 = β−0.8 with α = 0.05.

The much smaller standardized difference (Δ = 0.3) between two sites of vagus nerves sampling and pelvic nerves shown in Figure 14D can be confidently demonstrated due to the considerably larger number of available data points (121 pelvic vs. abd. vagus and 77 pelvic vs. cervical vagus pairwise measurements). Therefore, we can state that the dissimilarity between pelvic and abdominal vagus spatial architectures is much smaller than between pelvic and cervical vagus nerves (*p*-value = 0.0352). In other words, abdominal vagus samples resemble pelvic cross-sections to a higher degree than cervical vagus cross-sections.

## 4. Discussion

While many modern feature learning methods can directly classify biological images based on structural differences, the critical issue is the ability to quantify the specific architectural aspects of biological structures in order to relate them to a function or pathology. Neuroanatomy is one of the fields in which black-box image classifiers are undesirable, as the objective of the research is to link the image attributes to actual anatomical and physiological knowledge regarding cell and tissue organization, as opposed to simply sorting the images into predetermined categories. The approach presented here adds another module to our multi-step sample and data processing pipeline, which also includes the data acquisition and image segmentation modules described previously (Havton et al., 2021; Plebani et al., 2022).

We pursued the representation of the segmented unmyelinated axons in the TEM images of the peripheral nerve cross-sections as spatial point patterns not only to gain a better understanding of their neuroanatomy but also to express the observed differences in a quantitative manner, which would enable a variety of automated analysis tasks in the future, including automated image queries, image database retrieval, and biological image comparisons. While visual inspection of segmented images and their associated point patterns might provide some basic intuition regarding the spatial intensity, it is impossible to rely on the investigator's visual perception and judgments when examining more complex pattern characteristics such as local heterogeneous and anisotropic spatial features. Although global bulk measures of second-order spatial statistics, such as the *K*- and *L*-functions, help to represent and explore spatial interactions (randomness, inhibition, or clustering), they fail to capture local variations within biological structures. On the other hand, the local form of these spatial statistics functions generates yet another complex spatial pattern, leaving scientists with an equally tricky quantification problem. In this context, our analytical approach that captures differences between arrangements of any spatial distributions to form a visualizable embedding that enables straightforward comparison between complex structures provides a simple-to-use tool for neuroanatomists and computational neuroscientists.

There are at least three notable limitations associated with the demonstrated methods and their specific implementation. As previously stated, the claimed differences are relatively small and, despite being statistically significant, may be biologically unimportant. There is no reason to anticipate that the spatial arrangement would be dramatically altered in samples that do not represent a recognized disease. We realize that the value of the method would be more clearly demonstrated if the detected differences were associated with a specific biological mechanistic model, especially one associated with a disease or an abnormality. Although we lack such examples, we hope that researchers working on projects involving anatomical pathologies will be able to easily reproduce our methodology for quantifying observable differences in a biomedical context.

The second concern stems from the first: because there are no established alternative methods to quantify the spatial organization of axons, there is no way to validate the results by relating them to known physiology. This would indeed be a valuable exercise if the relevant nerves' physiology was already defined with sufficient precision. Unfortunately, this is not the case, so in the absence of sufficient data of this type, we have considered a number of well-established anatomical characteristics of these nerves that are consistent with our new study. For instance, our research aimed to distinguish between the cervical and abdominal vagus, and it revealed that the abdominal vagus and pelvic nerve share some similarities. This correlates with the higher prevalence of myelinated axons in the cervical vagus (Hulsebosch and Coggeshall, 1982; Prechtl and Powley, 1990), but it does not necessarily imply that the overall patterning of myelinated axons within each nerve type will be distinct. Physiological evidence of the type required to validate the current findings regarding sex differences in the vagus is also lacking. As many of the motor and sensory pathways supply sexually dimorphic targets, a sex difference may be anticipated for the pelvic nerve. However, in the present study, only samples from male rats were available.

The third concern relates to the orientation of biological structures. The anatomical rotational positions (orientations) of the analyzed fascicles were unknown (they were not recorded during sample processing), and we employed the post-hoc method described in Section 3.2 to identify the preferable orientation of the specimens. Although this method finds the rotations representing the smallest discrepancy between specimens, there is no guarantee that the identified orientations are biologically relevant. Sample alignment and orientation labeling is a broader problem in microscopy, not only affecting our analysis but also other techniques, such as multimodal imaging.

Despite these limitations, the presented method is an important contribution to the microscopy analytical toolbox. Notably, the availability of analytical tools is essential for the collection and evaluation of a large, comprehensive set of neuroanatomy and neuropathology data. As a result, the current scarcity of labeled and segmented images is attributable in part to the absence of an established analytic framework, casting doubt on the systematic value of acquiring comprehensive peripheral nerve data. We certainly hope that the conception and presentation of our method will inspire neuroanatomists to collect more data on peripheral nerves, resulting in broader quantitative anatomical studies. Importantly, our approach is simple to reproduce because it employs existing libraries for a popular statistical prototyping language.

Although we focused here on unmyelinated axons, the computational pipeline is applicable to multi-type point patterns and spatial research outside of neuroscience. This work demonstrates that the spatial architecture of unmyelinated axons in peripheral nerve cross-sections is neither uniform nor random but forms complex and rich arrangements. In order to simulate such a complicated spatial form, hybrid point processes are required. In the future, we plan to focus on spatial modeling and further classification of peripheral nerve cross-sections. The similarity (or dissimilarity) measure we established in this study will be the foundation for these modeling tasks.

## 5. Conclusions

In this report, we examined one of the key research problems in neuroanatomy, the fundamental description, measurement, and quantification of the spatial arrangement of axons in peripheral nerves such as the vagus and pelvic nerves (Hulsebosch and Coggeshall, 1982; Prechtl and Powley, 1990). This topic is significant not only from the basic neuroanatomical standpoint, but also due to the growing importance of peripheral nerve electrostimulation approaches, which rely on a precise understanding of the peripheral nerve architecture during the modeling and development phases (Pelot et al., 2020; Eiber et al., 2021). We believe that quantitative analysis, comparisons, and visualization of spatial arrangement can provide valuable insight to neuroanatomists, computational neuroscientists, and engineers working in the field of electrostimulation. We also believe that the presented method can be easily adapted to other biological fields, including spatial proteomics and genomics (Ji et al., 2020; Hickey et al., 2022).

## Data availability statement

The microscopy data associated with this study were collected as part of the Stimulating Peripheral Activity to Relieve Conditions (SPARC) program and are available at the SPARC Portal (RRID: SCR_017041) under CC-BY 4.0 license (Havton et al., 2022).

## Author contributions

BR conceived, planned, and supervised the spatial analysis study. AP contributed to the mathematical models. EP and MD preprocessed the microscopy data and designed the segmentation pipeline. TP and JK collected and processed the biological samples. TP provided neuroscience expertise and envisioned the quantitative peripheral nerve research. LH and NB collected and annotated the microscopy images. DJ curated the data and performed image preprocessing and mosaicing. AS and BR executed the study and co-wrote the manuscript with input from all the researchers. All authors contributed to the article and approved the submitted version.

## Footnotes

^1^At https://github.com/Banus/umf_unet.
